# Predictors of change in asthma-related quality of life: a longitudinal real-life study in adult asthmatics

**DOI:** 10.1007/s11136-022-03339-0

**Published:** 2023-01-03

**Authors:** Gilles Louis, Benoit Pétré, Florence Schleich, Halehsadat Nekoee Zahrei, Anne-Françoise Donneau, Monique Henket, Virginie Paulus, Françoise Guissard, Michèle Guillaume, Renaud Louis

**Affiliations:** 1 Department of Public Health, University of Liège, Liège, Belgium; 2 Department of Pneumology, University of Liège, GIGAI3 Liège, Belgium; 3 Department of Public Health, Biostatistics Unit, University of Liège, Liège, Belgium

**Keywords:** Asthma-related quality of life, Asthma control, BMI, Longitudinal study

## Abstract

**Purpose:**

Asthma negatively impacts health-related quality of life (HRQL). The objective is to investigate the longitudinal relationship between HRQL in asthma and disease control, demographic and clinical objective parameters in an adult population in real-life settings.

**Methods:**

We conducted a longitudinal study on adult asthmatics recruited from Liege University Hospital Asthma Clinic (Belgium) between 2011 and 2019. We selected those who had two visits and completed two patient-reported outcome measures (PROMs), the asthma control test (ACT) and the mini asthma quality of life questionnaire (AQLQ) (*n* = 290). AQLQ was the dependent variable. Demographic, functional and inflammatory characteristics, asthma control, and exacerbations were the independent variables. We applied generalized linear mixed models to identify the factors associated with change in AQLQ and its dimensions.

**Results:**

Median (IQR) time interval between the two visits was 7 (5–19) months. Overall, median (IQR) global AQLQ increased from 4.1 (3–5.1) to 4.6 (3.4–5.9) (*p* < 0.0001). All AQLQ dimensions significantly improved, apart the environmental one. AQLQ improved in patients who had both step-up and step-down pharmacological treatment as well as in patients reporting no change between the two visits. The fitted models indicated that change in ACT was the main predictor of change in AQLQ (*p* < 0.0001). A rise in 3 units in ACT predicted an improvement of 0.5 AQLQ (AUC-ROC = 0.85; *p* < 0.0001). Change in BMI inversely impacted global AQLQ (*p* < 0.01) and its activity dimension (*p* < 0.0001).

**Conclusion:**

Asthma control and BMI are key predictors of asthma quality of life acting in an opposite direction. AQLQ may improve without step-up in the pharmacological treatment.

**Supplementary Information:**

The online version contains supplementary material available at 10.1007/s11136-022-03339-0.

## Introduction

Globally, more than 350 million people are affected by asthma [1], a chronic airway disease characterized by reversible airway obstruction due to airway smooth muscle contraction, airway wall edema, and mucus hypersecretion causing increased resistance to airflow and difficulty in breathing [1, 2].

It is known that asthma negatively impacts the health-related quality of life (HRQL) [3, 4]. HRQL is a multidimensional concept that reflects the health and the effects of disease on the life from the patient’s perspective [5, 6]. Although objective clinical parameters are necessary to assess the disease, they are not sufficient to understand and evaluate how the patients perceive their health [7]. As a consequence, international guidelines have evolved to consider scientifically validated patient-reported outcome measures (PROMs) measuring asthma control and asthma-specific HRQL as important outcomes in asthma management [2, 8].

Understanding the predictors of asthma quality of life can be useful in designing interventions the purpose of which would be to improve health status of the patients and what really matters to them [9]. In this regard, many cross-sectional studies have already explored the factors associated with HRQL in asthma [6, 10, 11]. These studies showed that asthma control was the main factor associated with HRQL, although demographics such as social factors—gender [12, 13], age [6, 10], body mass index (BMI) [11, 14], level of education [15, 16] and occupation [16, 17]—were also found to contribute. In addition, lung function together with airway inflammatory parameters [11, 18, 19] including sputum neutrophils and fraction of exhaled nitric oxide (FeNO) were also shown to predict some dimensions of asthma quality of life, even after adjustment for asthma control and demographics [11].

With respect to longitudinal studies, clinical trials have shown improvement in asthma quality of life after pharmacological interventions including administration of inhaled corticosteroids (ICS) that was partly related to changes in lung function [20, 21]. Others have found, in a prospective observational study, an improvement of asthma quality of life in some non-eosinophilic asthmatics after decreasing the dose of ICS [22].

To the best of our knowledge, there has been no real-life longitudinal study in an asthmatic adult population, which investigated the relationship between asthma-related quality of life and asthma control, demographic, functional, and inflammatory features. Therefore, we have leveraged our large asthma clinic database to conduct a retrospective longitudinal study in a cohort of asthmatics who had been well characterized and seen at least at two visits. In this study, we sought to investigate how asthma quality of life may have changed over time and to which factors these changes might have been related.

## Methods

### Study design, setting, and participants

**Fig. 1:**
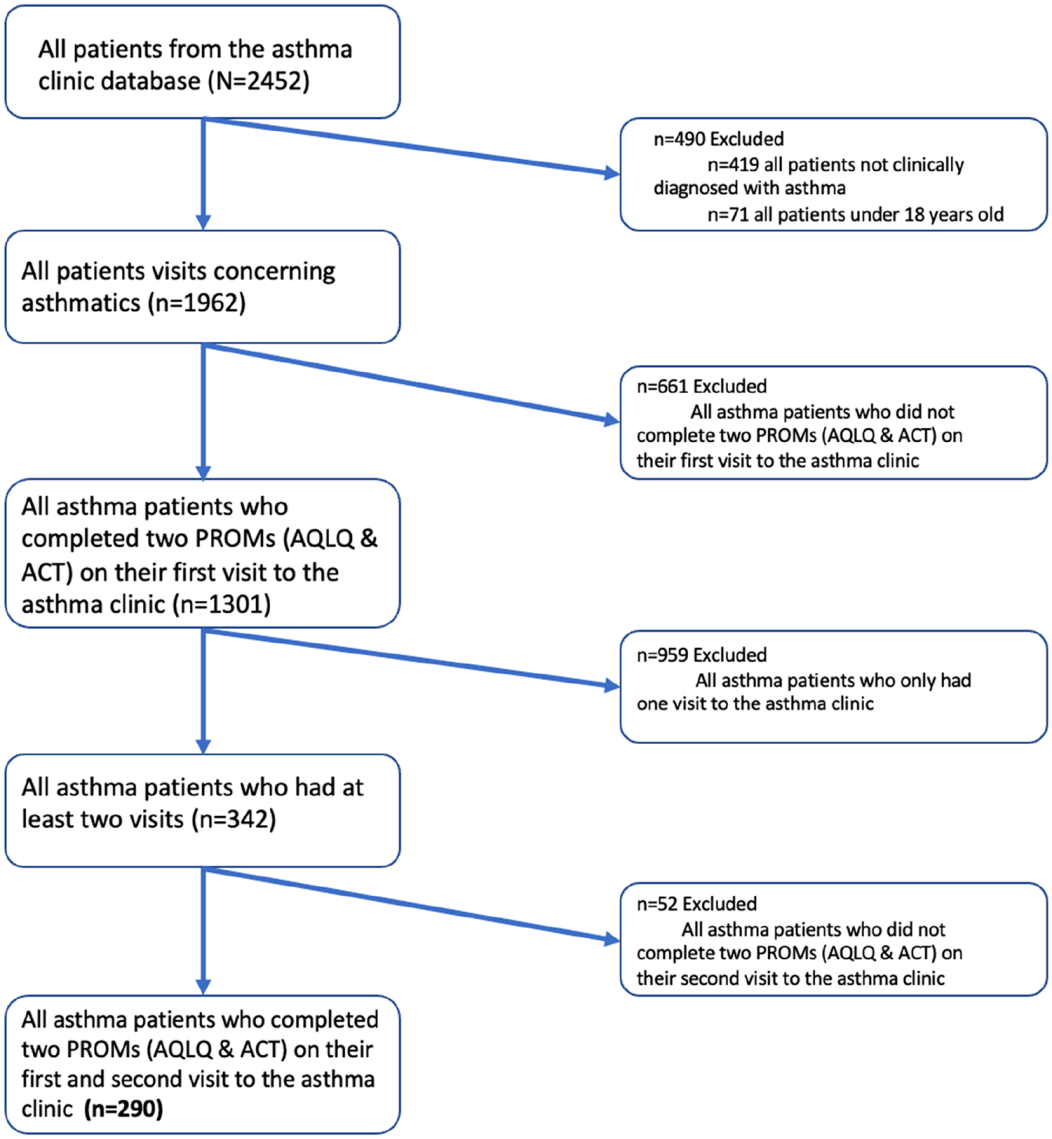
Flow chart of the patients selection process. *PROM* patient-reported outcome measure; *AQLQ* asthma quality of life questionnaire; *ACT* asthma control test

We conducted a retrospective longitudinal study on patients (≥ 18 years old at the first visit) recruited from the Liege University Hospital Asthma Clinic (Belgium) between 2011 and 2020. As described in our previous study [11], in accordance with the global initiative for asthma (GINA) criteria [2], the asthma diagnosis was based on the presence of typical symptoms (wheezing, breathlessness, chest tightness, and cough) combined with a 12% and 200 ml forced expiratory volume in 1 s (FEV1) reversibility after inhalation of 400 μg salbutamol, a β2 receptor agonist, and/or a provocative concentration of methacholine, a cholinergic agonist for muscarinic receptor, causing a 20% drop in FEV1 ≤ 16 mg/ml when FEV1 ≥ 70% predicted. We selected patients who had at least two visits at the asthma clinic and completed twice the Asthma Control Test [23] (ACT) and the Mini Asthma Quality of Life Questionnaire [24] (Mini AQLQ). When the patients had more than two visits, we systematically selected the first two visits (*n* = 290) (Fig. 1). Considering 3, 4, or 5 visits would have led to 20%, 45%, and 65% population attrition. As our intention was to include a large number of parameters in multiple regression analysis, we believed it was preferable to capture the larger number of patients as possible. In addition, selecting the first two visits increases the probability of unraveling relationship between pharmacological treatment and quality of life, as it is usually at the first visit that treatment is initiated or modified.

### Studied variables

All the studied variables described below were contained in an electronic database constructed and managed by a data manager of the pneumology department of CHU Liège (Belgium). All the variables were collected as part of the patient routine examination performed in the asthma clinic.

#### Asthma-related quality of life (dependent variable)

Asthma-related quality of life was measured using the Mini Asthma Quality of Life Questionnaire [24] (Mini AQLQ). This scientifically validated tool [24] includes 15 items divided into four dimensions: symptoms (5 items), activity limitation (4 items), emotional function (3 items), and environmental stimuli (3 items). The 15 items are scored on a seven-point Likert scale. The score for the questionnaire as a whole and the individual dimensions are simply averages of the responses to the questions within them [24]. Seven is the highest score in terms of asthma-related quality of life. The reliability of AQLQ for this study was calculated by intraclass correlation coefficient (ICC). The minimal clinically important difference (MCID)—the smallest difference in a quality of life score that the patient perceives as clinically important—is 0.5 for AQLQ [25].

#### Demographic and disease characteristics (independent variables)

Demographic and health characteristics were age, gender, atopy, smoking status, BMI, age of asthma onset, and treatment. As described in our previous study [11], atopy was defined by a positive IgE test (> 0.35 kU/L) to one or more common aeroallergens (grass pollen, tree pollen, cat, dog, molds, and house dust mite). Smoking status was divided into three categories: never-smoker, ex-smoker (quit smoking at least 6 months previously), and current smokers. Treatment was divided into four categories based on patient report at anamnesis: (1) no treatment; (2) short-acting beta agonists (SABA) alone as needed; (3) maintenance treatment including ICS + long-acting beta agonists (LABA) and/or leukotriene receptor antagonist (LTRA); and (4) any maintenance treatment combined with oral corticosteroid (OCS). None of the patients included in this study were treated with biologics.

Disease characteristics were asthma control, exacerbations, lung function, and systemic and airway inflammation. Asthma control was measured using the Asthma Control Test (ACT) [23], which consists of five questions related to symptomatology and activity limitation. Each question contains five propositions, each scored on a scale from 1 to 5. Studies have established cutoff scores for asthma that is well-controlled (ACT ≥ 20), not well-controlled (ACT ≤ 19) , and uncontrolled (ACT ≤ 15) [23, 26]. Exacerbations in the year prior to the visit were defined by at least a three-day course of OSC in non-OCS-treated patients and a quadrupling in dose for patients on maintenance OCS. Lung function testing was performed by spirometry (Spiro bank; MIR, Rome, Italy) in order to measure expiratory flow rates. A post-bronchodilator test was done for each patient, irrespective of their baseline FEV1 and FEV1/forced vital capacity (FVC) ratio, as a standard procedure in order to assess airway obstruction reversibility. Patients were administrated 400 μg of inhaled salbutamol via a metered-dose inhaler (Ventolin®), one puff at a time into the spacer, and spirometry was performed again 15 min later. Patients with baseline FEV1 ≥ 70% predicted were given a methacholine challenge test, as previously described [11]. Using tidal breathing, the subjects inhaled successive quadrupling methacholine concentrations from 0.06 to 16 mg/ml for one minute each; FEV1 was measured 30 and 90 s after each concentration. The test was stopped if FEV1 fell at least 20% from its baseline value. The provocative concentration of methacholine causing a 20% fall in FEV1 (PC20M) was calculated by linear interpolation from the last two points of the curve.

Inflammatory parameters included FeNO, sputum (airway secretion) cell counts (eosinophils and neutrophils), blood cell counts (eosinophils and neutrophils), and systemic markers C-reactive protein (CRP) and fibrinogen. FeNO was measured at a flow rate of 50 ml/s (NIOX; Aerocrine, Solna, Sweden) before spirometry. Sputum induction by inhalation of (hypertonic) saline and processing were performed as previously described [27]. CRP, fibrinogen, blood eosinophils, and neutrophils counts were determined by routine laboratory analysis at Liège University Hospital.

#### Care session organization

AQLQ and ACT were self-administrated to the patients during the same session of care under the supervision of a healthcare provider. The care session included lung function testing, sputum induction, FeNO measurement, and questionnaires. There were generally 5 to 10 min between the two questionnaires.

## Statistical analysis

In this study, quantitative variables were summarized based on medians and interquartile ranges (P25-P75), and qualitative variables were summarized using counts and percentages. The number and percentage of missing values were also reported in descriptive statistics tables.

To assess the effect of time on AQLQ and its dimensions (dependent variable), a univariate beta regression mixed model was fitted for AQLQ and its subscales that were each considered as the independent variables, with random effects for participant identification and a fixed effect for time. The same analysis was applied to estimate the effect of time on other factors (independent variables), in which different methods of generalized linear mixed models were fitted depending on the type of factors. To evaluate the effect of the demographic and disease characteristics on AQLQ and its dimensions over time, we first applied univariate beta regression mixed models where each factor was considered individually along with time and treatment changes group as fixed effects, as well as random effects for participant identification. Then, by incorporating all significant factors from the univariate models, a multivariate beta regression mixed model was fitted. To take into account the possibility of an important variable which could have not come out as significant because of confounding factors from the univariate analyses, we also fit the full model including all the variables.

Receiver operating characteristics (ROC) curve was constructed to determine the threshold of change in ACT that predicts a significant improvement of AQLQ (MCID: 0.5).

Finally, global AQLQ and its four dimensions were transformed to binary variables using cut point 6 which identifies an optimal asthma quality of life [28]. The dichotomous variables were created in such a way that AQLQ: 1 = AQLQ ≥ 6 / 0 = AQLQ < 6 (not optimal asthma quality of life). To evaluate how demographic and disease characteristics affect the binary AQLQ and its dimensions over time, univariate binary logistic mixed models were applied where in addition to time and treatment changes group as fixed effects, random effects for participant identification were also considered for each variable. The variables that were significant in the earlier models were used to fit multivariate binary logistic regression mixed models.

All statistical modeling was carried out using the statistical software R with a level of significance 0.05.

## Ethics

This study was approved by the Liège University Hospital ethics committee. Signed informed consent was obtained from patients as soon as they entered the asthma clinic. They agreed to allow their clinical data and the health outcomes they reported in the routine setting to be used for research purpose.

## Results

### Characteristics of the study population

Baseline demographic characteristics are presented in Table 1. Overall, 184 patients were female (63%) and 146 patients were atopic (50%). Median age was 54 years and median age at time of diagnosis was 37 years. Median time interval between the two visits was 7 months.

**Table 1:** Baseline demographic characteristics of patients (*n* = 290)

	Median (IQR)/percentage (frequency)	Missing value percentage (number)
Age (year)	54 (43–64)	0% (0)
Gender (female) % (n)	63% (184)	0% (0)
Onset of asthma (year)	37 (15–53)	17% (49)
Atopy (yes) % (n)	50% (146)	8% (24)
Time between the two visits (months)	7 (5–19)	0% (0)

### Comparison between baseline and follow-up visit

**Fig. 2:**
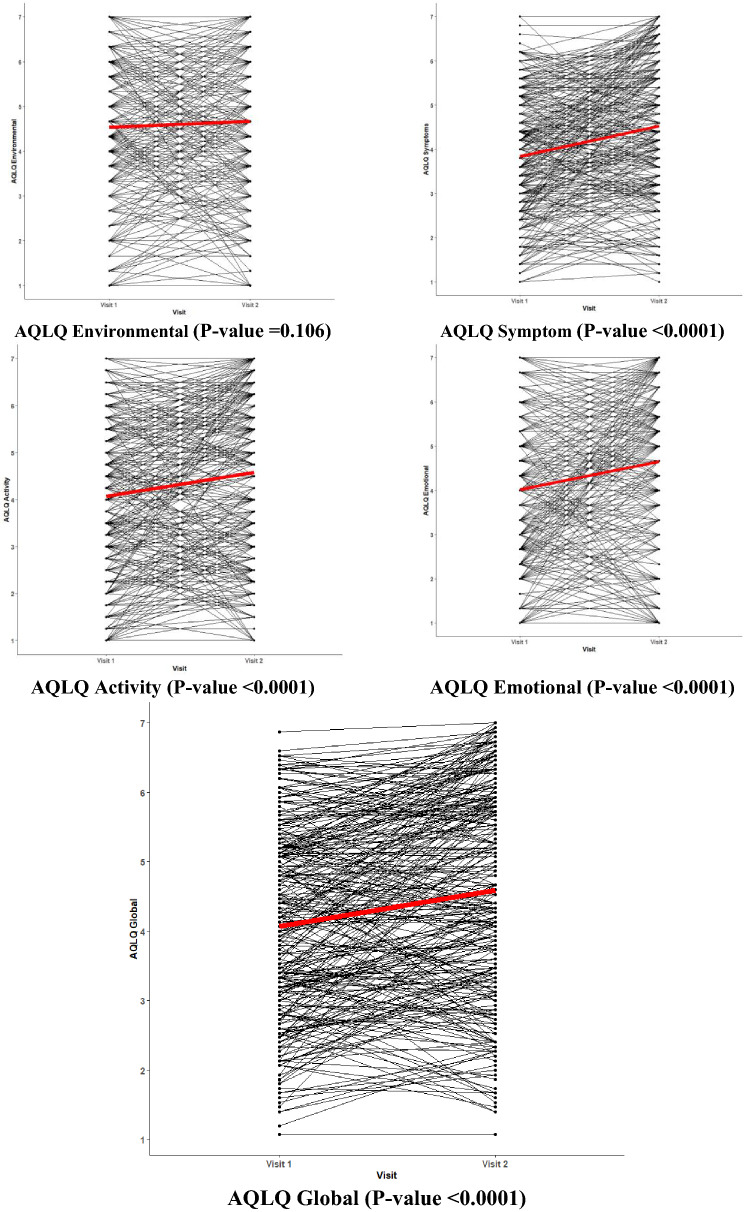
Spaghetti plot of individual changes in Global AQLQ and its subscales over time. *AQLQ* asthma quality of life questionnaire

The comparison between visit 1 and visit 2 is presented in Table 2. Median (IQR) global AQLQ increased from 4.07 (3–5.1) to 4.6 (3.4–5.9) (*p* < 0.0001) with significant improvement in all dimensions (*p* < 0.0001), except the environmental one (Fig. 2). An acceptable reliability was found for global AQLQ (ICC = 0.94), AQLQ symptom dimension (ICC = 0.88), AQLQ activity dimension (ICC = 0.92), AQLQ emotional dimension (ICC = 0.83), and AQLQ environmental dimension (ICC = 0.74). There was an increase in median asthma control (ACT) from 13 to 17 (*p* < 0.0001). The proportion of patients with exacerbation decreased from 58 to 47% (*p* < 0.01). Pre-bronchodilation median % predicted FEV1 increased from 78 to 80% (*p* < 0.01). Median pre and post-bronchodilation FEV1/FVC % rose from 71 to 74% (*p* < 0.0001) and from 75 to 76% (*p* < 0.05), respectively. Median FeNO levels decreased from 29 to 24 ppb (*p* < 0.0001) and median sputum eosinophil counts from 85.10^3^/g to 36.10^3^/g (*p* < 0.01). Median blood eosinophils decreased from 243/µl to 189/µl (*p* < 0.01).

**Table 2:** Comparison of patient characteristics between visit 1 and visit 2 (*n* = 290)

			Visit 1		Visit 2		
			Median (IQR)/percentage (frequency)	Missing value % (*n*)	Median (IQR)/percentage (frequency)	Missing value % (*n*)	Estimate (95% confidence interval)
Global AQLQ			4.07 (3.02–5.13)	0% (0)	4.6 (3.4–5.93)	0% (0)	0.41 (0.29–0.51)****
AQLQ Symptom			3.8 (2.8–5)	0.34% (1)	4.4 (3.4–5.8)	0% (0)	0.52 (0.39–0.65)****
AQLQ Activity			4.25 (2.75–5.25)	0.34% (1)	4.87 (3.25–6.19)	0% (0)	0.40 (0.27–0.54)****
AQLQ Emotional			4 (2.67–5.33)	0.34% (1)	5 (3.33–6.33)	0% (0)	0.45 (0.30–0.61)****
AQLQ Environmental			4.67 (3.33–6)	0.34% (1)	4.67 (3.33–6.25)	0% (0)	0.11 (− 0.02–0.25)
ACT			13 (9–17)	0% (0)	17 (11–22)	0% (0)	2.962 (2.305, 3.618)****
BMI (kg/m2)			26.3 (23.3–30.07)	0% (0)	26.4 (23.5–30.1)	0% (0)	0.062 (− 09, 021)
Exacerbation (Yes)			58% (151)	11% (29)	47% (98)	39% (89)	− 0.647 (− 1.143, − 0.181)**
Smoking Status		Non-smokers	52% (151)		52% (150)		
		Ex-smokers	32% (92)	1% (4)	35% (100)	1% (4)	− 0.492(− 1.49, 0.51)
		Current smokers	15% (43)		12% (36)		
FENO (ppb)			29 (16–65)	1% (3)	24 (15–43)	2% (5)	− 9.797 (− 14.667, − 4.926)****
Sputum neutrophils (10^3^/g)			853 (249–3060)	12% (35)	793 (300–2073)	29% (85)	− 0.056 (− 0.223, 0.112)
Sputum eosinophils (10^3^/g)			85 (4–532)	12% (35)	36 (4–148)	29% (85)	− 0.135 (− 0.288, 0.018)
Blood neutrophils (µL)			4212 (3289–5398)	1% (3)	4327 (3366–5371.5)	4% (11)	− 0.041 (− 0.135, 0.052) &
Blood eosinophils (µL)			243 (131–430)	1% (3)	189 (91–384)	3% (8)	− 0.179 (− 0.265, − 0.094)****
Total IgE (KU/L)			125 (41–383)	5% (15)	127 (44–364)	21% (60)	− 0.011 (− 0.149, 0.129)
Fibrinogen (g/l)			3 (3–4)	10% (29)	3 (2.83–3.92)	13% (38)	− 0.043 (− 0.145, 0.058)
CRP (mg/l)			2.44 (1.08–5.91)	6% (17)	2.30 (1–4.9)	12% (36)	− 0.056 (− 0.197, 0.085)
FEV1 pre (%)			78 (58–89)	0.34% (1)	80 (65–93.75)	0% (0)	2.678 (0.814,4.541) **
FEV1 post (%)			85 (68–98)	0.34% (1)	86 (70.75–97)	1% (2)	0.393 (− 1.38, 2.168)
FVC pre (%)			90 (75–102)	0% (0)	89 (77.25–101.75)	0% (0)	0.165 (− 1.651, 1.981)
FVC post (%)			94 (78–106)	0.34% (1)	94 (78.5–106)	1% (3)	-1.408 (− 3.142, 0.326)
FEV1/FVC pre			71 (64–79)	0% (0)	74 (66–80)	0% (0)	1.931 (0.964, 2.898)****
FEV1/FVC post			75 (67–81)	0.34% (1)	76 (68.75–83)	1% (2)	1.017 (0.040, 1.993) *
Asthma Treatment	No treatment		8% (22)	0% (0)	3% (10)		6.32 (− 3.44, 9.21)****
	SABA alone		20% (59)		7% (19)		
	ICS + LABA and/or LTRA		53% (154)		80% (233)		
	Maintenance treatment + OCS		19% (55)		10% (28)		

*AQLQ* asthma quality of life questionnaire; *ACT* asthma control test; *FENO* Fraction of exhaled nitric oxide; *FEV1* Forced expiratory volume in 1 s; *FVC* Forced vital capacity; *SABA* short-acting beta agonist; *ICS* Inhaled corticosteroids; *LABA* Long-acting beta agonists; *LTRA* Leukotriene receptor antagonist; *OCS* Oral corticosteroids

SABA alone: 14 patients had OCS as maintenance + SABA as needed

*Significant at the *p* < 0.05 level; **Significant at the *p* < 0.01 level; ***Significant at the *p* < 0.001 level; ****Significant at the *p* < 0.0001 level. &: *p* = 0.08

With respect to treatment, the proportion of patients treated by SABA alone fell significantly from 20 to 7% (*p* < 0.0001), while the proportion of patients receiving a maintenance treatment including ICS/LABA and/or LTRA rose from 53 to 80% (*p* < 0.05). Overall, the mean (SEM) beclomethasone equivalent increased from 1086 (± 60) μg/d to 1288 (± 56) μg/d (*p* < 0.0001). The proportion of patients with OCS maintenance decreased from 19% at baseline to 10% at follow-up. Globally, pharmacological treatment changes between baseline and follow-up were classified into 6 categories. Improvement in global AQLQ and all its dimension, except the environmental one, was observed in all categories of treatment changes, except in no treatment to SABA (Table 3).

**Table 3:** Comparison of AQLQ and its dimensions in six groups defined by treatment changes between visits 1 and 2 (*n* = 290)

Variables	Visit	No change (*n* = 61)	No treatment to SABA (*n* = 5)	No treatment or SABA to ICS/LABA or step-up ICS (*n* = 131)	Treated or not treated to OCS (*n* = 5)	Step down ICS/LTRA (*n* = 47)	Stop OCS (*n* = 41)	Global *p* value
		Median (IQR)	Median (IQR)	Median (IQR)	Median (IQR)	Median (IQR)	Median (IQR)	
Global AQLQ	First	4.07 (3.27–4.93)	5.33 (5.20–6.47)	4.27 (3.13–5.20)	3.20 (2.80–4.0)	4.13 (3.23–5.36)	3.13(2.33–4.67)	0.009
	Second	4.87 (3.73–6.0)**	5.73 (5.47–6.07)	4.80 (3.4–5.9)****	4.13 (3.73–5.73)*	5.13 (3.4–5.9)**	3.93(2.93–4.40)**	
AQLQ Symptom	First	3.6 (3.15–4.65)	5.6 (3.8–6.0)	4.0 (3.0–4.9)	3.4 (2.4–4.2)	4.2 (3.0–5.1)	3.0(2.2–4.0)	0.029
	Second	4.8 (3.8–5.8)**	5.6 (5.2–5.8)	4.6 (3.3–6.0)****	3.8 (3.6–4.6)*	4.4 (3.5–6.0)**	3.8(3.0–4.6)***	
AQLQ Activity	First	4.50 (3.25–5.56)	5.50 (4.75–6.75)	4.25 (3.0–5.50)	2.75 (2.0–2.75)	4.50 (3.0–5.25)	2.75(2.0–3.75)	< 0.001
	Second	5.25 (4.0–6.25)**	5.25 (5.0–5.75)	5.0 (3.12–6.25)**	4.0 (3.25–5.75)*	5.25 (3.6–6.2)**	3.50(2.75–4.50)***	
AQLQ Emotional	First	4.17 (2.67–5.33)	5.0 (2.67–6.0)	4.33 (2.67–5.5)	3.0 (2.67–4.0)	4.33 (2.5–6.0)	3.0(2.0–4.67)	0.014
	Second	5.0 (3.67–6.67)**	5.33 (4.33–6.33)	5.0 (3.33–6.0)****	5.0 (3.67–6.33)*	5.33 (3.0–6.67)**	4.0(2.67–5.0)**	
AQLQ Environmental	First	4.83 (3.33–6.0)	6.0 (3.67–6.33)	4.33 (3.33–6.00)	4.67 (3.67–6.33)	4.33 (3.0–5.67)	4.33(3.67–5.33)	0.210
	Second	4.67 (3.33–6.33)	5.67( 3.33–7.0)	4.67 (3.67–6.33)	5.67 (4.0–7.0)**	4.67 (3.17–6.17)	4.0(3.33–5.33)	

*AQLQ* asthma quality of life questionnaire; *SABA* short-acting beta agonist; *ICS* Inhaled corticosteroids; *LABA* Long-acting beta agonists; *LTRA* Leukotriene receptor antagonist; *OCS* Oral corticosteroids

Global *p* value represents the differences between treatment groups over time in the generalized mixed model when no changes level was the reference

The significant values presented the changes in global AQLQ and its dimensions over time in each group individually

*Significant at the *p* < 0.05 level; **Significant at the *p* < 0.01 level; ***Significant at the *p* < 0.001 level; ****Significant at the *p* < 0.0001 level

There was 48% of asthmatics who had an improvement in AQLQ ≥ 0.5 (MCID) , while only 17% had a deterioration with a decrease in AQLQ ≥ 0.5.

### The factors associated with change in asthma-related quality of life (continuous variable) over time

**Fig. 3:**
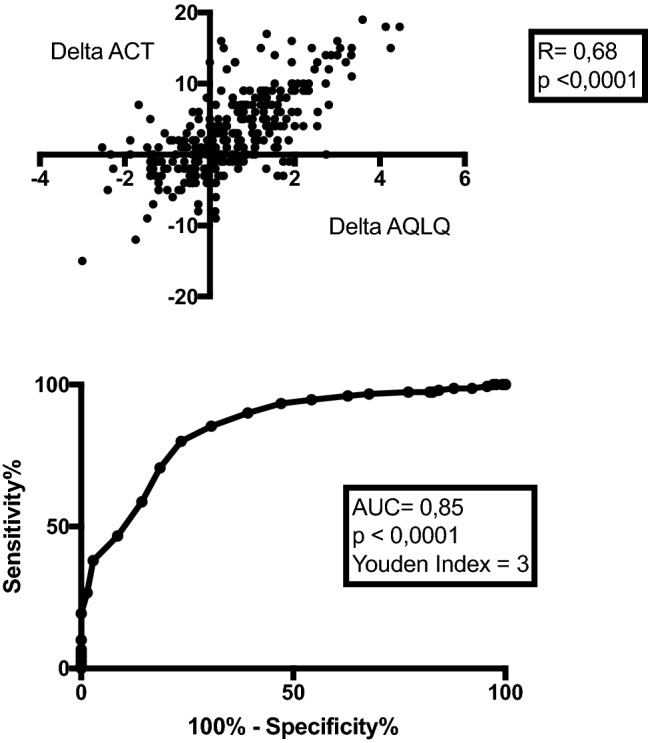
Relationship between delta ACT and delta AQLQ (upper panel). ROC curve showing change in ACT as a predictor of a change of 0.5 in AQLQ (lower panel). *AQLQ* asthma quality of life questionnaire; *ACT* asthma control test; *ROC* receiver operating characteristic

Results of univariate beta regression mixed models are given in online supplement Table S1, where results for each independent variables after fixing the effect for time and treatment changes group were provided. All the significant factors in the univariate beta regression mixed models for global AQLQ and/or its 4 dimensions were investigated in a multivariate beta regression mixed model (Table 4). Only ACT had a significant impact on global AQLQ (Fig. 3 upper panel) and on its 4 dimensions (*p* < 0.0001 for all), which increased when ACT increased. When drawing a ROC curve, we found that a change in ACT of 3 was the best threshold to predict an improvement of 0.5 AQLQ (AUC = 0.85; *p* < 0.0001) (Fig. 3 lower panel). The global AQLQ significantly decreased with increasing BMI (*p* < 0.01). Change in activity dimension of AQLQ was associated with change in BMI and decreased as BMI increased (*p* < 0.0001) (Table 4). When fitting the full model including all variables, only ACT and BMI were still significantly associated with global AQLQ in the multivariable model (data not shown).

**Table 4:** Multivariate beta regression mixed model for factors associated with AQLQ and its subscales over time

Factors	Global AQLQ		Symptom dimension		Activity dimension		Emotional dimension		Environmental dimension	
	Estimate (std.error)	*P* Value	Estimate (std.error)	*P* Value	Estimate (std.error)	*P* Value	Estimate (std.error)	*P* Value	Estimate (std.error)	*P* Value
ACT	**0.18 (0.01)**	< 0.0001	**0.21 (0.01)**	< 0.0001	**0.21 (0.009)**	< 0.0001	**0.194 (0.01)**	< 0.0001	**0.111 (0.015)**	< 0.0001
BMI (kg/m^2^)	− **0.03 (0.01)**	0.009	− 0.01(0.01)	0.079	− **0.05 (0.01)**	< 0.0001			-0.02(0.02)	0.263
Onset of asthma	0.0004 (0.002)	0.853					0.005 (0.004)	0.221	0.006 (0.004)	0.146
Sputum eosinophils (10^3^/g)	− 0.002 (0.002)	0.437	− 0.001 (0.002)	0.599	0.001 (0.002)	0.570	− 0.01 (0.003)	0.174		
Blood eosinophils (µL)	0.001 (0.01)	0.885	− 0.0001 (0.01)	0.989	− 0.001 (0.012)	0.913	0.002 (0.018)	0.885	0.011 (0.018)	0.539
Fibrinogen (g/l)	0.011 (0.05)	0.828	–	–	0.007 (0.055)	0.887				
FEV1 pre (%)	0.044 (0.026)	0.097	0.012 (0.03)	0.638	0.01 (0.03)	0.736	0.102 (0.045)	0.025	0.0001 (0.01)	0.992
FEV1 post (%)	− 0.03 (0.03)	0.324	− 0.008 (0.02)	0.724	− 0.009 (0.028)	0.74	− 0.08 (0.044)	0.049	0.003 (0.013)	0.807
FEV1/FVC pre	− 0.06 (0.03)	0.035	− 0.007 (0.03)	0.794	− 0.034 (0.032)	0.282	− 0.131 (0.05)	0.008		
FEV1/FVC post	0.051 (0.030)	0.093	0.007 (0.03)	0.788	0.037 (0.031)	0.227	0.136 (0.049)	0.006		
No change	Reference									
No treatment to SABA	0.53 (0.44)	0.234	− 0.21 (0.35)	0.551	0.05 (0.37)	0.892	1.08 (0.78)	0.166	0.769 (0.768)	0.316
No treatment to ICS-LABA	− 0.11 (0.12)	0.369	0.02 (0.11)	0.83	− 0.14 (0.37)	0.262	− 0.178 (0.21)	0.395	− 0.131 (0.22)	0.561
Treated to OCS	0.17 (0.38)	0.64	0.35 (0.31)	0.26	− 0.22 (0.34)	0.52	0.02 (0.653)	0.974	0.718 (0.71)	0.31
Step down ICS/LTRA	− 0.23 (0.15)	0.12	− 0.07 (0.13)	0.62	− 0.29 (0.15)	0.052	0.03 (0.251)	0.897	− 0.25 (0.27)	0.359
Stop OCS	− 0.09 (0.18)	0.60	0.18 (0.15)	0.22	− 0.33 (0.17)	0.055	− 0.196 (0.29)	0.509	0.097 (0.314)	0.757
Time	− 0.01(0.08)	0.913	0.05 (0.07)	0.517	0.055 (0.08)	0.513	0.025 (0.13)	0.845	− 0.22 (0.12)	0.059

In bold factors that have a *p* value < 0.001

*AQLQ* asthma quality of life questionnaire; *ACT* asthma control test; *FEV1* Forced expiratory volume in 1 s; *FVC* Forced vital capacity; *SABA* short-acting beta agonist; *ICS* Inhaled corticosteroids; *LABA* Long-acting beta agonists; *LTRA* Leukotriene receptor antagonist; *OCS* Oral corticosteroids

The empty cell indicates that the factor was not significantly associated with AQLQ, in the univariate analysis

### The factors associated with obtaining an optimal asthma-related quality of life (≥ 6; binary variable) over time

Global AQLQ and its four dimensions were also transformed to binary variables using cut point 6. The proportion of patients with global AQLQ ≥ 6 rose from 8% in visit 1 to 22% in visit 2 (p < 0.0001) (Table 5).

**Table 5:** Descriptive statistics of converted binary AQLQ (cutoff 6) in visits 1 and 2 (*n* = 290)

	Levels	Visit 1	Visit 2	Odds ratio (95% confidence interval)
		Percentage (frequency)	Percentage (frequency)	
Global AQLQ	Low (< 6)	92% (266)	78% (225)	234.85 (489.2–1144.6)****
	High (≥ 6)	8% (24)	22% (65)	
AQLQ Symptom	Low (< 6)	93% (269)	76% (219)	5.629 (2.91–10.89)****
	High (≥ 6)	7% (21)	24% (71)	
AQLQ Activity	Low (< 6)	84% (243)	73% (211)	2.389 (1.486–3.994)**
	High (≥ 6)	16% (47)	27% (79)	
AQLQ Emotional	Low (< 6)	79% (230)	67% (193)	2.818 (1.725–4.852)***
	High (≥ 6)	21% (60)	33% (97)	
AQLQ Environmental	Low (< 6)	72% (210)	69% (199)	1.320 (0.846–2.079)
	High (≥ 6)	28% (80)	31% (91)	

Results of univariate binary logistic mixed models are given in online supplement Table S2, where results for each independent variables after fixing the effect for time and treatment changes group were provided. All the significant factors in the univariate binary logistic mixed models for converted binary global AQLQ and/or its 4 dimensions were considered in a multivariate binary logistic regression mixed model (Table 6). Increasing ACT over time increased the probability of achieving a high level of global AQLQ and its 4 dimensions over time (*p* < 0.0001). One unit increase in BMI (*p* < 0.01) as well as stopping OCS (*p* < 0.05) compared to no changes level decreased the odds ratio of being in the high level of global AQLQ. Likewise, increasing BMI decreased the probability of achieving a high level in activity dimension of AQLQ (*p* < 0.01). Finally, a higher FeNO associated with a greater probability of achieving a high level in the activity dimension of AQLQ (*p* < 0.01) (Table 6).

**Table 6:** Multivariate binary logistic regression mixed model for factors associated with converted binary AQLQ and its subscales over time

Factors	Global AQLQ		Symptom dimension		Activity dimension		Emotional dimension		Environmental dimension	
	Odds ratio	*P* value	Odds ratio	*P* value	Odds ratio	*P* value	Odds ratio	*P* value	Odds ratio	*P* value
ACT	**1.924**	< 0.0001	**1.678**	< 0.0001	**1.556**	< 0.0001	**1.446**	< 0.0001	**1.206**	< 0.0001
BMI (kg/m^2^)	**0.881**	0.013			**0.866**	0.003				
FENO (ppb)					**1.012**	0.008				
Sputum neutrophils (10^3^/g)							1.007	0.348		
Sputum eosinophils (10^3^/g)	0.995	0.750	1.005	0.713			0.987	0.297		
Blood eosinophils (µL)	0.842	0.054	0.883	0.134			0.981	0.728		
FEV1 pre (%)	1.007	0.855	1.067	0.168	0.814	0.198	1.038	0.761	0.996	0.897
FEV1 post (%)	0.984	0.702	0.931	0.131	1.268	0.141	0.966	0.777	1.008	0.795
FEV1/FVC pre					1.247	0.201	0.991	0.954		
FEV1/FVC post					0.786	0.165	1.004	0.978		
No change	Reference									
No treatment to SABA	4.385	0.226	1.864	0.564	0.873	0.905	3.333	0.358	4.038	0.300
No treatment to ICS-LABA	0.741	0.555	0.853	0.734	0.942	0.891	0.871	0.748	0.878	0.734
Treated to OCS	1.708	0.704	0.308	0.476	0.739	0.827	1.641	0.725	2.209	0.545
Step down ICS/LTRA	0.382	0.129	0.339	0.068	0.810	0.704	1.119	0.828	0.833	0.699
Stop OCS	0.133	0.028	0.713	0.661	0.382	0.267	1.074	0.913	0.669	0.538
Time	1.347	0.472	2.006	0.067	0.748	0.409	0.861	0.630	0.627	0.134

In bold, factors that have a *p* value < 0.01

*AQLQ* asthma quality of life questionnaire; *ACT* asthma control test; *FENO* Fraction of exhaled nitric oxide; *FEV1* Forced expiratory volume in 1 s; Forced vital capacity; *SABA* short-acting beta agonist; *ICS* Inhaled corticosteroids; *LABA* Long-acting beta agonists; *LTRA* Leukotriene receptor antagonist; *OCS* Oral corticosteroids

The empty cell indicates that the factor was not significantly associated with AQLQ, in the univariate analysis

## Discussion

In this longitudinal real-life study, it turned out that asthma-related quality of life and all its dimensions, except the environmental one, improved after a first passage at the asthma clinic irrespective of the pharmacological treatment changes. We further identified changes in asthma control and in BMI as key predictors of change in AQLQ. On the whole cohort, this improvement was obtained with modest increase in the daily dose of ICS reaching 19%. The proportion of patients with optimal global AQLQ increased from 8 to 20%.

### Predictors of changes in AQLQ

Many cross-sectional studies have already demonstrated that asthma control is a major factor associated with asthma quality of life [6, 10, 11]. In addition, Chen et al. who conducted a prospective observational cohort study (TENOR study) [29] showed that asthma control was an independent predictor of HRQL in asthma both at baseline and after a 12 months follow-up. Here, using real-world data, we go one step further by demonstrating that changes in asthma control as reflected by changes in ACT, is the main predictor of change in AQLQ. Furthermore, by constructing ROC curve, we found that an increase of three in ACT was the best threshold to predict an increase of 0.5 in AQLQ, which represent the MCID.

Another interesting result that emerged from the current study is the fact that change in BMI was found to be an independent predictor of change in global AQLQ and its activity dimension over time. The impact of high BMI on asthma quality of life and on its activity dimension had already been reported in cross-sectional studies [11, 14, 30]. Dramatic changes in BMI obtained after Bariatric surgery were found to improve asthma control and asthma quality of life [31]. None of our patients had undergone bariatric surgery between the two visits. To the best of our knowledge, we are reporting here for the first time that change in BMI, independent of a bariatric surgery, may be an independent factor of asthma quality of life after adjustment for asthma control. We believe this is an important finding as it would suggest that reducing BMI by providing nutritional counseling and promoting regular physical activity in asthmatics may be a way to improve quality of life independent of asthma control.

In this study, changes in lung function and airway eosinophilic inflammation were not found as independent factors associated with changes in AQLQ. We believe however that these factors were determinants in improving asthma control, as previously demonstrated in both cross-sectional and longitudinal studies [32, 33]. Therefore, asthma control mediates the effect of improving lung function and reducing inflammation on asthma quality of life. A surprising finding of the current study is the fact that a rise in FeNO is an independent predictor of the probability of achieving an optimal AQLQ in its activity dimension. This is in keeping with our recently published cross-sectional study [11], where we demonstrated that FeNO level was an independent predictor positively associated the activity dimension of AQLQ. High FeNO has traditionally been seen as a bad outcome reflecting an eosinophilic inflammation [34]. Roberts et al.[35] showed that quality of life declines with increasing FeNO as a result of pollen allergen exposure. Our population is far from being exclusively atopic, thereby limiting the relationship between allergen exposure and subsequent rise in FeNO leading to deterioration in asthma quality of life. The reason why rise in FeNO might increase the probability of achieving an excellent AQLQ in its activity dimension in our study remains to be investigated but it is worth noting that NO is a potent mediator of vasodilation, a physiological process critical in skeletal muscle O2 supply. Our multivariate statistical analysis revealed that patient perspective measured through PROM may actually question the interpretation of FeNO level in asthma and suggest that FeNO and eosinophils, although being correlated, may actually have different effects on asthma quality of life.

### Influence of pharmacological treatment changes

One important finding of our study is the fact that asthma quality of life improves between the first passage at the asthma clinic and the follow-up visit, whichever the pharmacological treatment change. If the proportion of patients on SABA alone decreased from 20 to 6% and the proportion of patients with maintenance treatment ICS/LABA or LTRA increased from 50 to 83% between baseline and follow-up providing a pharmacological rationale for the improvement in quality of life, there was also decreased treatment burden in other patients with either stopping maintenance OCS or reducing the dose of ICS. This suggests that some patients are over treated, and that decreasing corticoids exposure might be beneficial in some circumstances. The dedicated chest physicians to our asthma clinic are using markers of T2 inflammation including sputum eosinophils, FeNO, and blood eosinophils to initiate or adjust the dose of ICS [36–38]. Therefore, the existence/ persistence of T2 biomarkers is an impetus to start or increase the dose of ICS but a decrease in corticoids may be proposed to the patients who combine low eosinophils and low FeNO, which may result in an improvement of asthma quality of life [22]. Why quality of life also improved in those patients with no treatment change may be explained by several factors and is in keeping with a previous study [39]. First, as it is a real-life retrospective study, we cannot assume that adherence to treatment was optimal at baseline. We can anticipate that time dedicated to the patient and investigations by health care professionals during the passage at the asthma clinic (approximately 60–80 min) would have increased health literacy and adhesion to the pharmacological treatment strategy [40]. Therefore, an improved adherence to usual treatment and/or a better handling of aerosol devices by the patient might have led to a better asthma control and quality of life [41]. Second, we have also to consider non-pharmacological factors such as an engagement of the patient toward a better nutrition or regular physical activity [42, 43] as it is generally advised by the health care professionals working in our asthma clinic. Our finding is supported by the demonstration that providing patient with information about the disease, particularly by increasing patients’ knowledge of how to treat their symptoms, may improve asthma control and quality of life [44]. Indeed, this reinforcement of knowledge on the disease and on the techniques of taking bronchodilators is part of therapeutic patient education recognized as contributing to an improvement of quality of life, although this practice is still not formalized and institutionalized in Belgium [45].

### Strengths and limitations of the study

One strength of this study is the fact that our cohort encompassed a wide variety of asthmatics with whom healthcare professionals have to deal in their everyday clinical practice including moderate-to-severe asthma but also some milder form of the disease. This gives confidence that our finding may be of relevance to a global asthma population and there is increased recognition of the importance of real-life studies to support the findings of randomized control trials (RCT) [46].Other strengths are the extensive clinical characterization of the patients and the use of scientifically validated PROMs (ACT and Mini-AQLQ). Nevertheless, the current study has several limitations. First, the absence of sociodemographic characteristics—such as the level of instruction or the occupation—is regrettable when it is known that they may influence HRQL [6, 10]. Second, we limited our analysis by comparing only two visits and it would have been interesting to see whether quality of life further improved with recurrent passages at asthma clinic. Third, among those who attended twice, some did not complete ACT and AQLQ at the second visit (52/342 = 15%) (see Fig. 1). A fourth limitation is the fact that our analysis did not include several comorbidities—such as rhinosinusitis or gastroesophageal reflux—known to impact the asthma quality of life [47, 48].

## Conclusion

This longitudinal study demonstrates that asthma control is the leading factor of asthma-related quality of life over time, and thereby justify that it is the key element of asthma management. This study also shows that some demographic characteristics such as BMI must be considered in the asthma management and that asthma quality of life may sometimes improve without increasing pharmacological treatment burden.

## Supplementary Information

Below is the link to the electronic supplementary material.

Supplementary file1 (PDF 550 kb)

## Data Availability

The datasets generated and/or analyzed during the current study are not publicly available due to the privacy of certain patient data, but are available from the corresponding author on reasonable request.

## Abbreviations

ACT: Asthma control test
AQLQ: Asthma quality of life questionnaire
BMI: Body mass index
FENO: Fraction of exhaled nitric oxide
FEV1: Forced expiratory volume in 1 s
FVC: Forced vital capacity
GINA: Global initiative for asthma
ICS: Inhaled corticosteroids
LABA: Long-acting beta agonists
LTRA: Leukotriene receptor antagonist
OCS: Oral corticosteroids
PC20M: Provocative concentration of methacholine causing a fall in FEV1 of 20%
RCT: Randomized control trial
SABA: Short-acting beta agonists
