# Supplementary material for: Predictors of change in asthma-related quality of life: a longitudinal real-life study in adult asthmatics
Source: Qual Life Res. 2023 Jan 3;32(5):1507–20. doi: 10.1007/s11136-022-03339-0 (PMC10123047; doi:10.1007/s11136-022-03339-0)
Supplement: Supplementary file 1 — Supplementary file1 (PDF 550 kb) [file 11136_2022_3339_MOESM1_ESM.pdf]

Supplementary Table 1 (S1). Univariate beta regression mixed models on AQLQ and its dimensions based on independent variables individually, with time and treatment changes group as fixed effect

S1.1 Effect of ACT on AQLQ and its dimensions

**Parameters**	**Global AQLQ**		**AQLQ Symptom**		**AQLQ Activity**		**AQLQ Emotional**		**AQLQ Environmental**	
	**Estimate (std.error)**	**P-value**	**Estimate (std.error)**	**P-value**	**Estimate (std.error)**	**P-value**	**Estimate (std.error)**	**P-value**	**Estimate (std.error)**	**P-value**
ACT	**0.19(0.01)**	<0.0001	**0.21(0.01)**	<0.0001	**0.21(0.01)**	<0.001	**0.21(0.01)**	<0.0001	**0.12(0.01)**	<0.0001
Time	-0.05(0.06)	0.32	0.07(0.06)	0.24	-0.11(0.07)	0.11	0.001(0.08)	0.99	-0.23(0.08)	0.008
No change	Reference									
No treatment to SABA	0.42(0.31)	0.16	0.23(0.32)	0.46	0.13(0.38)	0.72	0.60(0.48)	0.21	0.95(0.57)	0.10
No treatment, SABAtoICS/LABA	-0.08(0.09)	0.39	-0.05(0.1)	0.61	-0.23(0.12)	0.07	-0.12(0.16)	0.46	0.02(0.19)	0.91
Treated or not treated to OCS	0.17(0.30)	0.56	0.08(0.31)	0.81	-0.45(0.37)	0.22	0.39(0.48)	0.54	0.95(0.58)	0.09
Step down ICS/LTRA	-0.13(0.12)	0.30	-0.11(0.13)	0.37	-0.12(0.16)	0.45	-0.08(0.21)	0.66	-0.25(0.24)	0.29
Stop OCS	-0.09(0.13)	0.48	0.03(0.14)	0.81	-0.43(0.16)	0.009	-0.19(0.21)	0.37	0.13(0.25)	0.61

S1.2 Effect of age on AQLQ and its dimensions

Parameters	Global AQLQ		AQLQ Symptom		AQLQ Activity		AQLQ Emotional		AQLQ Environmental	
	Estimate (std.error)	P-value	Estimate (std.error)	P-value	Estimate (std.error)	P-value	Estimate (std.error)	P-value	Estimate (std.error)	P-value
Age (Year)	0.004(0.004)	0.364	0.01(0.004)	0.09	-0.003(0.005)	0.62	0.004(0.006)	0.47	0.007(0.005)	0.19
Time	0.52(0.07)	<0.0001	0.68(0.09)	<0.0001	0.52(0.09)	<0.001	0.63(0.09)	<0.001	0.13(0.08)	0.13
No change	Reference									
No treatment to SABA	0.99(0.58)	0.08	0.78(0.58)	0.18	0.85(0.66)	0.19	1.22(0.74)	0.09	1.23(0.69)	0.07
No treatment, SABAtoICS/LABA	0.06(0.19)	0.75	-0.03(0.19)	0.86	-0.21(0.22)	0.33	-0.09(0.24)	0.69	0.04(0.23)	0.87
Treated or not treated to OCS	-0.28(0.58)	0.62	-0.43(0.57)	0.45	-0.92(0.66)	0.16	-0.21(0.73)	0.78	0.63(0.69)	0.35
Step down ICS/LTRA	-0.01(0.24)	0.98	0.01(0.24)	0.95	-0.01(0.28)	0.96	0.04(0.30)	0.89	-0.16(0.28)	0.57
Stop OCS	-0.75(0.25)	0.002	-0.67(0.25)	0.007	-1.14(0.28)	<0.0001	-0.90(0.32)	0.004	-0.29(0.31)	0.33

S1.3 Effect of sex on AQLQ and its dimensions

Parameters	Global AQLQ		AQLQ Symptom		AQLQ Activity		AQLQ Emotional		AQLQ Environmental	
	Estimate (std.error)	P-value	Estimate (std.error)	P-value	Estimate (std.error)	P-value	Estimate (std.error)	P-value	Estimate (std.error)	P-value
Sex (Female)	**-0.34(0.15)**	0.025	-0.23(0.15)	0.133	**-0.37(0.17)**	0.03	-0.31(0.19)	0.11	**-0.49(0.18)**	0.007
Time	0.53(0.07)	<0.0001	0.69(0.08)	<0.001	0.51(0.08)	<0.001	0.63(0.09)	<0.0001	0.13(0.08)	0.11
No change	Reference									
No treatment to SABA	1.10(0.57)	0.05	0.92(0.57)	0.11	0.86(0.65)	0.19	1.32(0.73)	0.07	1.41(0.68)	0.04
No treatment, SABAtoICS/LABA	-0.08(0.19)	0.67	-0.05(0.19)	0.79	-0.23(0.22)	0.29	0.11(0.24)	0.63	0.01(0.22)	0.96
Treated or not treated to OCS	-0.28(0.57)	0.62	-0.40(0.57)	0.48	-0.96(0.65)	0.14	-0.20(0.73)	0.77	0.64(0.68)	0.35
Step down ICS/LTRA	0.01(0.24)	0.94	0.01(0.24)	0.95	0.03(0.27)	0.91	0.06(0.30)	0.85	-0.14(0.28)	0.63
Stop OCS	-0.81(0.25)	0.001	-0.71(0.25)	0.005	-1.22(0.28)	<0.001	-0.96(0.32)	0.002	-0.38(0.3)	0.19

S1.4 Effect of BMI on AQLQ and its dimensions

Parameters	Global AQLQ		AQLQ Symptom		AQLQ Activity		AQLQ Emotional		AQLQ Environmental	
	**Estimate (std.error)**	**P-value**	**Estimate (std.error)**	**P-value**	**Estimate (std.error)**	**P-value**	**Estimate (std.error)**	**P-value**	**Estimate (std.error)**	**P-value**
BMI (kg/m^2)	**-0.04(0.01)**	0.003	**-0.04(0.08)**	0.01	**-0.07(0.02)**	<0.001	-0.02(0.02)	0.24	**-0.04(0.02)**	0.03
Time	0.53(0.07)	<0.001	0.91(0.57)	<0.001	0.52(0.09)	<0.001	0.64(0.09)	<0.001	0.14(0.08)	0.10
No change	Reference									
No treatment to SABA	1.08(0.57)	0.06	0.91(0.57)	0.11	0.85(0.63)	0.18	1.29(0.73)	0.07	1.36(0.69)	0.05
No treatment, SABAtoICS/LABA	-0.02(0.19)	0.91	-0.001(0.19)	0.99	-0.14(0.21)	0.49	-0.08(0.24)	0.75	0.07(0.23)	0.77
Treated or not treated to OCS	-0.23(0.57)	0.69	-0.36(0.57)	0.53	-0.88(0.63)	0.16	-0.16(0.73)	0.82	0.71(0.69)	0.30
Step down ICS/LTRA	-0.04(0.24)	0.86	-0.03(0.24)	0.91	-0.04(0.26)	0.87	0.02(0.30)	0.95	-0.21(0.29)	0.47
Stop OCS	-0.66(0.25)	0.008	-0.58(0.25)	0.02	-1.0(0.27)	0.003	-0.85(0.32)	0.007	-0.21(0.30)	0.49

S1.5 Effect of smoking status on AQLQ and its dimensions

Parameters	Global AQLQ		AQLQ Symptom		AQLQ Activity		AQLQ Emotional		AQLQ Environmental	
	Estimate (std.error)	P-value	Estimate (std.error)	P-value	Estimate (std.error)	P-value	Estimate (std.error)	P-value	Estimate (std.error)	P-value
Non-smokers	Reference									
Ex-Smokers	0.16(0.14)	0.28	0.18(0.15)	0.23	0.02(0.17)	0.92	0.09(0.19)	0.61	**0.34(0.17)**	0.06
Current Smokers	-0.01(0.20)	0.97	-0.27(0.21)	0.19	-0.01(0.23)	0.97	-0.33(0.25)	0.19	**0.64(0.24)**	0.007
Time	0.52(0.07)	<0.0001	-0.26(0.21)	<0.001	0.52(0.08)	<0.0001	0.64(0.09)	<0.0001	0.14(0.08)	0.08
No change	Reference									
No treatment to SABA	0.99(0.57)	0.08	0.68(0.09)	0.13	0.81(0.66)	0.22	1.29(0.73)	0.08	1.18(0.68)	0.08
No treatment, SABAtoICS/LABA	-0.07(0.19)	0.69	0.86(0.57)	0.91	-0.19(0.22)	0.37	-0.07(0.24)	0.76	0.001(0.23)	0.99
Treated or not treated to OCS	-0.29(0.58)	0.61	-0.02(0.19)	0.49	0.94(0.66)	0.15	-0.16(0.73)	0.83	0.58(0.68)	0.39
Step down ICS/LTRA	-0.03(0.24)	0.87	-0.38(0.57)	0.94	-0.005(0.27)	0.98	0.02(0.30)	0.94	-0.17(0.28)	0.54
Stop OCS	-0.78(0.25)	0.001	-0.66(0.25)	0.008	-1.16(0.29)	<0.0001	-0.88(0.32)	0.006	-0.36(0.29)	0.22

S1.6 Effect of atopy on AQLQ and its dimensions

Parameters	Global AQLQ		AQLQ Symptom		AQLQ Activity		AQLQ Emotional		AQLQ Environmental	
	Estimate (std.error)	P-value	Estimate (std.error)	P-value	Estimate (std.error)	P-value	Estimate (std.error)	P-value	Estimate (std.error)	P-value
Atopy (Yes)	0.02(0.15)	0.86	0.12(0.15)	0.41	0.10(0.17)	0.55	-0.09(0.19)	0.61	-0.17(0.18)	0.35
Time	0.51(0.07)	<0.0001	0.68(0.08)	<0.0001	0.48(0.09)	<0.0001	0.60(0.10)	<0.0001	0.12(0.08)	0.5
No change	Reference									
No treatment to SABA	1.19(0.57)	0.03	1.09(0.57)	0.06	0.91(0.65)	0.1	1.41(0.73)	0.06	1.41(0.69)	0.04
No treatment, SABAtoICS/LABA	0.12(0.19)	0.54	0.15(0.19)	0.43	0.08(0.22)	0.74	0.12(0.25)	0.62	0.21(0.24)	0.37
Treated or not treated to OCS	-0.13(0.57)	0.82	-0.23(0.57)	0.68	-0.87(0.65)	0.18	-0.01(0.73)	0.98	0.81(0.69)	0.24
Step down ICS/LTRA	0.01(0.25)	0.97	0.06(0.25)	0.1	-0.03(0.29)	0.91	0.08(0.32)	0.81	-0.17(0.30)	0.58
Stop OCS	-0.72(0.26)	0.006	-0.59(0.26)	0.02	-1.15(0.31)	0.003	-0.89(0.33)	0.007	-0.33(0.32)	0.30

S1.7 Effect of onset of asthma on AQLQ and its dimensions

Parameters	Global AQLQ		AQLQ Symptom		AQLQ Activity		AQLQ Emotional		AQLQ Environmental	
	Estimate (std.error)	P-value	Estimate (std.error)	P-value	Estimate (std.error)	P-value	Estimate (std.error)	P-value	Estimate (std.error)	P-value
Onset of asthma	**0.07(0.003)**	0.036	0.01(0003)	0.184	0.004(0.004)	0.27	**0.01(0.004)**	0.02	**0.01(0.004)**	0.009
Time	0.38(0.09)	<0.0001	0.49(0.12)	<0.0001	0.41(0.11)	0.0001	0.50(0.13)	<0.0001	0.04(0.11)	0.74
No change	Reference									
No treatment to SABA	1.86(0.72)	0.009	1.79(0.71)	0.01	1.67(0.85)	0.05	2.39(0.95)	0.01	1.71(0.91)	0.06
No treatment, SABAtoICS/LABA	-0.03(0.21)	0.87	0.09(0.21)	0.63	-0.19(0.25)	0.44	-0.10(0.27)	0.71	-0.01(0.26)	0.97
Treated or not treated to OCS	-0.93(0.65)	0.15	-0.79(0.66)	0.23	-1.91(0.77)	0.01	-1.13(0.86)	0.19	0.27(0.82)	0.74
Step down ICS/LTRA	0.09(0.25)	0.69	0.21(0.25)	0.41	0.04(0.30)	0.88	0.26(0.33)	0.44	-0.15(0.32)	0.64
Stop OCS	-0.72(0.29)	0.01	-0.68(0.29)	0.02	-1.09(0.34)	0.001	-0.83(0.37)	0.03	-0.19(0.36)	0.58

S1.8 Effect of FeNO on AQLQ and its dimensions

Parameters	Global AQLQ		AQLQ Symptom		AQLQ Activity		AQLQ Emotional		AQLQ Environmental	
	Estimate (std.error)	P-value	Estimate (std.error)	P-value	Estimate (std.error)	P-value	Estimate (std.error)	P-value	Estimate (std.error)	P-value
FENO (ppb)	-0.001(0.001)	0.59	-0.002(0.001)	0.12	0.001(0.002)	0.35	-0.001(0.002)	0.41	0.001(0.01)	0.56
Time	0.55(0.07)	<0.0001	0.69(0.09)	<0.001	0.57(0.08)	<0.0001	0.64(0.1)	<0.001	0.17(0.09)	0.04
No change	Reference									
No treatment to SABA	1.03(0.58)	0.07	0.86(0.58)	0.14	0.79(0.65)	0.22	1.26(0.73)	0.08	1.32(0.69)	0.06
No treatment, SABAtoICS/LABA	-0.06(0.19)	0.76	-0.01(0.19)	0.97	-0.24(0.22)	0.27	-0.07(0.24)	0.75	0.01(0.23)	0.96
Treated or not treated to OCS	-0.28(0.58)	0.62	-0.41(0.58)	0.48	-0.96(0.65)	0.14	-0.19(0.73)	0.79	0.65(0.69)	0.35
Step down ICS/LTRA	-0.05(0.24)	0.85	-0.02(0.24)	0.94	-0.06(0.27)	0.83	0.01(0.31)	0.97	-0.23(0.28)	0.42
Stop OCS	-0.77(0.25)	0.002	-0.67(0.25)	0.007	-1.21(0.28)	<0.0001	-0.91(0.32)	0.004	-0.32(0.30)	0.28

S1.9 Effect of sputum neutrophils on AQLQ and its dimensions

Parameters	Global AQLQ		AQLQ Symptom		AQLQ Activity		AQLQ Emotional		AQLQ Environmental	
	Estimate (std.error)	P-value	Estimate (std.error)	P-value	Estimate (std.error)	P-value	Estimate (std.error)	P-value	Estimate (std.error)	P-value
Sputum neutrophils (10^3/g)	0.001(0.002)	0.48	0.002(0.002)	0.30	-0.01(0.02)	0.72	0.003(0.002)	0.24	-0.001(0.002)	0.96
Time	0.53(0.08)	<0.0001	0.66(0.09)	<0.001	0.55(0.11)	<0.001	0.65(0.11)	<0.0001	0.18(0.09)	0.06
No change	Reference									
No treatment to SABA	1.11(0.67)	0.09	0.71(0.67)	0.29	1.11(0.75)	0.14	1.49(0.84)	0.08	1.25(0.81)	0.12
No treatment, SABAtoICS/LABA	-0.07(0.21)	0.73	0.03(0.21)	0.89	-0.30(0.24)	0.19	-0.14(0.26)	0.61	-0.05(0.25)	0.84
Treated or not treated to OCS	-0.09(0.6)	0.88	-0.11(0.60)	0.84	-0.79(0.68)	0.24	-0.06(0.76)	0.93	0.73(0.72)	0.31
Step down ICS/LTRA	-0.002(0.26)	0.99	0.06(0.26)	0.82	-0.09(0.29)	0.75	0.07(0.33)	0.94	-0.25(0.31)	0.42
Stop OCS	-0.68(0.28)	0.01	-0.58(0.28)	0.04	-1.21(0.31)	0.0001	-0.91(0.35)	0.009	-0.11(0.34)	0.75

S1.10 Effect of sputum eosinophils on AQLQ and its dimensions

Parameters	Global AQLQ		AQLQ Symptom		AQLQ Activity		AQLQ Emotional		AQLQ Environmental	
	**Estimate (std.error)**	**P-value**	**Estimate (std.error)**	**P-value**	**Estimate (std.error)**	**P-value**	**Estimate (std.error)**	**P-value**	**Estimate (std.error)**	**P-value**
Sputum eosinophils (10^3/g)	**-0.01(0.002)**	0.0001	**-0.01(0.004)**	<0.001	**-0.01(0.003)**	0.009	**-0.01(0.004)**	<0.001	-0.01(0.003)	0.06
Time	0.47(0.08)	<0.0001	0.59(0.09)	<0.001	0.48(0.11)	<0.0001	0.59(0.11)	<0.001	0.13(0.09)	0.16
No change	Reference									
No treatment to SABA	1.01(0.66)	<0.0001	0.62(0.67)	0.35	1.01(0.75)	0.18	1.41(0.83)	0.09	1.18(0.81)	0.14
No treatment, SABAtoICS/LABA	-0.05(0.21)	0.13	0.06(0.21)	0.77	-0.26(0.24)	0.26	-0.11(0.26)	0.68	-0.03(0.25)	0.91
Treated or not treated to OCS	-0.06(0.59)	0.81	-0.07(0.59)	0.90	-0.77(0.67)	0.25	-0.01(0.75)	0.98	0.75(0.72)	0.30
Step down ICS/LTRA	-0.05(0.26)	0.91	0.02(0.26)	0.94	-0.12(0.29)	0.68	0.02(0.32)	0.95	-0.27(0.31)	0.38
Stop OCS	-0.67(0.27)	0.85	-0.56(0.28)	0.04	-1.19(0.31)	0.0001	-0.89(0.34)	0.01	-0.10(0.33)	0.76

S1.11 Effect of blood neutrophils on AQLQ and its dimensions

Parameters	Global AQLQ		AQLQ Symptom		AQLQ Activity		AQLQ Emotional		AQLQ Environmental	
	Estimate (std.error)	P-value	Estimate (std.error)	P-value	Estimate (std.error)	P-value	Estimate (std.error)	P-value	Estimate (std.error)	P-value
Blood neutrophils (µL)	0.005(0.005)	0.31	0.003(0.006)	0.64	0.002(0.006)	0.71	0.005(0.007)	0.45	0.009(0.006)	0.17
Time	0.53(0.07)	<0.0001	0.68(0.09)	<0.0001	0.52(0.08)	<0.0001	0.64(0.09)	<0.0001	0.14(0.08)	0.09
No change	Reference									
No treatment to SABA	1.04(0.58)	0.07	0.88(0.58)	0.13	0.79(0.65)	0.22	1.25(0.73)	0.08	1.33(0.69)	0.06
No treatment, SABAtoICS/LABA	-0.07(0.19)	0.71	-0.05(0.19)	0.79	-0.22(0.22)	0.31	-0.12(0.25)	0.63	0.03(0.23)	0.87
Treated or not treated to OCS	-0.33(0.58)	0.57	-0.42(0.58)	0.47	-0.98(0.65)	0.13	-0.27(0.74)	0.72	0.58(0.69)	0.40
Step down ICS/LTRA	-0.03(0.24)	0.88	-0.01(0.24)	0.95	-0.03(0.27)	0.89	-0.005(0.31)	0.98	-0.18(0.29)	0.52
Stop OCS	-0.74(0.25)	0.003	-0.65(0.25)	0.009	-1.14(0.28)	<0.0001	-0.91(0.32)	0.004	-0.25(0.31)	0.39

S1.12 Effect of blood eosinophils on AQLQ and its dimensions

Parameters	Global AQLQ		AQLQ Symptom		AQLQ Activity		AQLQ Emotional		AQLQ Environmental	
	Estimate (std.error)	P-value	Estimate (std.error)	P-value	Estimate (std.error)	P-value	Estimate (std.error)	P-value	Estimate (std.error)	P-value
Blood eosinophils (µL)	**-0.06(0.01)**	<0.0001	**-0.07(0.01)**	<0.0001	**-0.07(0.02)**	<0.0001	**-0.07(0.02)**	<0.0001	**-0.03(0.02)**	0.04
Time	0.49(0.07)	<0.0001	0.63(0.08)	<0.0001	0.47(0.09)	<0.0001	0.59(0.09)	<0.001	0.13(0.08)	0.14
No change	Reference									
No treatment to SABA	0.99(0.58)	0.08	0.84(0.57)	0.15	0.74(0.65)	0.26	1.20(0.73)	0.10	1.31(0.69)	0.06
No treatment, SABAtoICS/LABA	-0.04(0.19)	0.85	-0.01(0.19)	0.96	-0.18(0.22)	0.41	0.08(0.24)	0.75	0.04(0.23)	0.85
Treated or not treated to OCS	-0.31(0.58)	0.59	-0.42(0.57)	0.46	-0.99(0.65)	0.13	-0.25(0.73)	0.73	0.65(0.69)	0.34
Step down ICS/LTRA	-0.07(0.24)	0.77	-0.05(0.24)	0.82	-0.08(0.27)	0.77	-0.04(0.31)	0.88	-0.19(0.29)	0.51
Stop OCS	-0.73(0.25)	0.003	-0.64(0.25)	0.01	-1.14(0.28)	<0.0001	-0.89(0.32)	0.005	-0.25(0.30)	0.41

S1.13 Effect of total IgE on AQLQ and its dimensions

Parameters	Global AQLQ		AQLQ Symptom		AQLQ Activity		AQLQ Emotional		AQLQ Environmental	
	Estimate (std.error)	P-value	Estimate (std.error)	P-value	Estimate (std.error)	P-value	Estimate (std.error)	P-value	Estimate (std.error)	P-value
Total IgE (KU/L)	0.0001(0.0001)	0.49	0.0001(0.0001)	0.198	0.0001(0.0001)	0.96	0.0001(0.0001)	0.75	0.0001(0.0001)	0.19
Time	0.52(0.08)	<0.0001	0.68(0.09)	<0.001	0.51(0.09)	<0.0001	0.61(0.11)	<0.0001	0.12(0.09)	0.15
No change	Reference									
No treatment to SABA	1.07(0.56)	0.06	0.93(0.56)	0.09	0.82(0.65)	0.21	1.29(0.73)	0.08	1.34(0.68)	0.04
No treatment to ICS	-0.01(0.19)	0.93	0.01(0.19)	0.94	-0.15(0.23)	0.50	-0.04(0.26)	0.86	0.04(0.24)	0.85
Treated to OCS	-0.25(0.56)	0.66	-0.36(0.56)	0.53	-0.92(0.65)	0.16	-0.17(0.73)	0.81	0.67(0.68)	0.33
Step down ICS/LTRA	-0.02(0.24)	0.93	-0.03(0.25)	0.91	-0.01(0.28)	0.96	0.04(0.32)	0.89	-0.18(0.29)	0.53
Stop OCS	-0.77(0.26)	0.003	-0.67(0.26)	0.009	-1.13(0.29)	0.0001	-0.91(0.33)	0.005	-0.4(0.31)	0.19

S1.14 Effect of fibrinogen on AQLQ and its dimensions

Parameters	Global AQLQ		AQLQ Symptom		AQLQ Activity		AQLQ Emotional		AQLQ Environmental	
	Estimate (std.error)	P-value	Estimate (std.error)	P-value	Estimate (std.error)	P-value	Estimate (std.error)	P-value	Estimate (std.error)	P-value
Fibrinogen (g/l)	**-0.15(0.07)**	0.03	-0.13(0.08)	0.08	**-0.19(0.08)**	0.02	-0.11(0.09)	0.22	-0.06(0.08)	0.41
Time	0.55(0.07)	<0.001	0.70(0.09)	<0.001	0.59(0.09)	<0.0001	0.66(0.10)	<0.0001	0.16(0.09)	0.06
No change	Reference									
No treatment to SABA	1.02(0.58)	0.08	0.91(0.58)	0.12	0.73(0.65)	0.26	1.27(0.74)	0.08	1.29(0.69)	0.06
No treatment, SABAtoICS/LABA	-0.03(0.20)	0.88	0.04(0.20)	0.86	-0.19(0.23)	0.38	-0.04(0.26)	0.86	0.01(0.24)	0.98
Treated or not treated to OCS	-0.17(0.59)	0.77	-0.26(0.59)	0.66	-0.79(0.66)	0.23	-0.12(0.75)	0.87	0.68(0.71)	0.33
Step down ICS/LTRA	0.04(0.25)	0.86	0.12(0.25)	0.63	-0.001(0.28)	0.99	0.13(0.32)	0.68	-0.21(0.30)	0.48
Stop OCS	-0.75(0.26)	0.004	-0.60(0.26)	0.02	-1.15(0.29)	0.001	-0.91(0.33)	0.006	-0.40(0.31)	0.20

S1.15 Effect of FEV1 pre (%) on AQLQ and its dimensions

Parameters	Global AQLQ		AQLQ Symptom		AQLQ Activity		AQLQ Emotional		AQLQ Environmental	
	Estimate (std.error)	P-value	Estimate (std.error)	P-value	Estimate (std.error)	P-value	Estimate (std.error)	P-value	Estimate (std.error)	P-value
FEV1 pre (%)	**0.02(0.003)**	<0.0001	**0.02(0.003)**	<0.0001	**0.03(0.003)**	<0.0001	**0.03(0.003)**	<0.0001	**0.01(0.003)**	<0.001
Time	0.45(0.06)	<0.0001	0.62(0.08)	<0.0001	0.43(0.08)	<0.0001	0.56(0.09)	<0.0001	0.09(0.08)	0.26
No change	Reference									
No treatment to SABA	0.72(0.55)	0.19	0.58(0.56)	0.29	0.41(0.61)	0.50	0.91(0.68)	0.186	1.14(0.68)	0.09
No treatment to ICS	-0.009(0.18)	0.96	0.01(0.19)	0.95	-0.14(0.20)	0.50	-0.03(0.23)	0.876	0.07(0.22)	0.75
Treated or not treated to OCS	0.03(0.55)	0.95	-0.12(0.56)	0.83	-0.56(0.61)	0.35	0.15(0.69)	0.827	0.86(0.68)	0.20
Step down ICS/LTRA	-0.04(0.23)	0.85	-0.03(0.23)	0.90	-0.02(0.25)	0.92	0.004(0.29)	0.99	-0.19(0.28)	0.49
Stop OCS	-0.53(0.24)	0.02	-0.46(0.25)	0.06	-0.87(0.27)	0.001	-0.65(0.30)	0.03	-0.15(0.29)	0.62

S1.16 Effect of FEV1 post (%) on AQLQ and its dimensions

Parameters	Global AQLQ		AQLQ Symptom		AQLQ Activity		AQLQ Emotional		AQLQ Environmental	
	**Estimate (std.error)**	**P-value**	**Estimate (std.error)**	**P-value**	**Estimate (std.error)**	**P-value**	**Estimate (std.error)**	**P-value**	**Estimate (std.error)**	**P-value**
FEV1 post (%)	**0.02(0.003)**	<0.001	**0.02(0.003)**	<0.001	**0.03(0.003)**	<0.001	**0.02(0.003)**	<0.001	**0.01(0.003)**	<0.001
Time	0.51(0.07)	<0.001	0.66(0.08)	<0.0001	0.49(0.08)	<0.001	0.61(0.09)	<0.001	0.13(0.08)	0.12
No change	Reference									
No treatment to SABA	0.73(0.55)	0.19	0.63(0.57)	0.27	0.41(0.62)	0.51	0.94(0.67)	0.18	1.13(0.68)	0.09
No treatment to ICS/LABA	0.008(0.18)	0.96	0.02(0.19)	0.89	-0.11(0.20)	0.59	-0.01(0.23)	0.94	0.08(0.23)	0.71
Treated or not treated to OCS	-0.05(0.55)	0.92	-0.21(0.57)	0.71	-0.66(0.62)	0.28	0.05(0.69)	0.94	0.81(0.68)	0.23
Step down ICS/LTRA	-0.02(0.23)	0.92	-0.01(0.24)	0.97	-0.01(0.26)	0.98	0.03(0.29)	0.92	-0.18(0.28)	0.51
Stop OCS	-0.51(0.24)	0.04	-0.45(0.25)	0.07	-0.83(0.27)	0.002	-0.63(0.30)	0.04	-0.15(0.29)	0.61

S1.17 Effect of FVC pre (%) on AQLQ and its dimensions

Parameters	Global AQLQ		AQLQ Symptom		AQLQ Activity		AQLQ Emotional		AQLQ Environmental	
	Estimate (std.error)	P-value	Estimate (std.error)	P-value	Estimate (std.error)	P-value	Estimate (std.error)	P-value	Estimate (std.error)	P-value
FVC pre (%)	**0.02(0.003)**	<0.001	**0.02(0.003)**	<0.001	**0.03(0.003)**	<0.001	**0.02(0.003)**	<0.001	**0.01(0.003)**	<0.001
Time	0.52(0.06)	<0.001	0.68(0.08)	<0.0001	0.51(0.08)	<0.001	0.63(0.09)	<0.001	0.13(0.08)	0.12
No change	Reference									
No treatment to SABA	0.85(0.56)	0.13	0.72(0.57)	0.20	0.54(0.61)	0.38	1.07 (0.7)	0.12	1.22(0.68)	0.07
No treatment, SABAtoICS/LABA	0.02(0.18)	0.92	0.03(0.19)	0.86	-0.09(0.20)	0.64	-0.01(0.23)	0.96	0.08(0.23)	0.70
Treated or not treated to OCS	-0.14(0.56)	0.80	-0.28(0.56)	0.62	-0.76(0.61)	0.21	-0.05(0.70)	0.94	0.75(0.68)	0.27
Step down ICS/LTRA	-0.07(0.23)	0.76	-0.05(0.24)	0.84	-0.07(0.26)	0.78	-0.02(0.29)	0.94	-0.21(0.28)	0.45
Stop OCS	-0.54(0.24)	0.02	-0.49(0.25)	0.05	-0.87(0.27)	0.001	-0.69(0.31)	0.02	-0.16(0.29)	0.59

S1.18 Effect of FVC post (%) on AQLQ and its dimensions

Parameters	Global AQLQ		AQLQ Symptom		AQLQ Activity		AQLQ Emotional		AQLQ Environmental	
	Estimate (std.error)	P-value	Estimate (std.error)	P-value	Estimate (std.error)	P-value	Estimate (std.error)	P-value	Estimate (std.error)	P-value
FVC post (%)	**0.02(0.003)**	<0.001	**0.02(0.003)**	<0.001	**0.03(0.003)**	<0.0001	**0.02(0.004)**	<0.001	**0.01(0.003)**	<0.001
Time	0.54(0.07)	<0.001	0.59(0.08)	<0.0001	0.54(0.08)	<0.0001	0.65(0.09)	<0.0001	0.15(0.08)	0.06
No change	Reference									
No treatment to SABA	0.82(0.56)	0.14	0.71(0.57)	0.21	0.48(0.63)	0.44	1.05(0.71)	0.14	1.18(0.68)	0.08
No treatment, SABAtoICS/LABA	-0.005(0.19)	0.97	0.01(0.19)	0.95	-0.13(0.21)	0.54	-0.04(0.24)	0.86	0.08(0.23)	0.73
Treated or not treated to OCS	-0.26(0.56)	0.64	-0.38(0.57)	0.50	-0.94(0.62)	0.13	-0.18(0.71)	0.79	0.68(0.68)	0.32
Step down ICS/LTRA	-0.06(0.23)	0.78	-0.04(0.24)	0.88	-0.07(0.26)	0.79	-0.02(0.29)	0.95	-0.21(0.28)	0.45
Stop OCS	-0.57(0.25)	0.02	-0.51(0.25)	0.04	-0.90(0.27)	<0.001	-0.72(0.31)	0.02	-0.18(0.29)	0.54

S1.19 Effect of FEV1/FVC pre on AQLQ and its dimensions

Parameters	Global AQLQ		AQLQ Symptom		AQLQ Activity		AQLQ Emotional		AQLQ Environmental	
	Estimate (std.error)	P-value	Estimate (std.error)	P-value	Estimate (std.error)	P-value	Estimate (std.error)	P-value	Estimate (std.error)	P-value
FEV1/FVC pre	**0.02(0.005)**	<0.0001	**0.02(0.005)**	<0.0001	**0.02(0.006)**	<0.001	**0.03(0.007)**	<0.001	0.007(0.006)	0.23
Time	0.48(0.07)	<0.0001	0.64(0.08)	<0.0001	0.47(0.09)	<0.001	0.57(0.09)	<0.001	0.12(0.08)	0.15
No change	Reference									
No treatment to SABA	0.98(0.57)	0.08	0.83(0.57)	0.15	0.74(0.65)	0.25	1.19(0.72)	0.09	1.32(0.69)	0.06
No treatment, SABAtoICS	-0.06(0.19)	0.73	-0.04(0.19)	0.84	-0.21(0.22)	0.33	-0.09(0.24)	0.68	0.03(0.23)	0.88
Treated or not treated to OCS	-0.05(0.57)	0.92	-0.18(0.58)	0.75	-0.72(0.65)	0.27	0.10(0.72)	0.88	0.75(0.69)	0.2
Step down ICS/LTRA	0.02(0.24)	0.93	0.03(0.24)	0.89	0.03(0.27)	0.89	0.08(0.29)	0.79	-0.17(0.29)	0.55
Stop OCS	-0.67(0.25)	0.006	-0.58(0.25)	0.01	-1.07(0.28)	<0.001	-0.80(0.31)	0.01	-0.26(0.30)	0.39

S1.20 Effect of FEV1/FVC post on AQLQ and its dimensions

Parameters	Global AQLQ		AQLQ Symptom		AQLQ Activity		AQLQ Emotional		AQLQ Environmental	
	Estimate (std.error)	P-value	Estimate (std.error)	P-value	Estimate (std.error)	P-value	Estimate (std.error)	P-value	Estimate (std.error)	P-value
FEV1/FVC post	**0.02(0.005)**	<0.0001	**0.02(0.006)**	<0.0001	**0.03(0.006)**	<0.001	**0.03(0.007)**	<0.001	0.007(0.006)	0.22
Time	0.49(0.07)	<0.0001	0.65(0.08)	<0.0001	0.47(0.08)	<0.001	0.56(0.09)	<0.0001	0.12(0.08)	0.13
No change	Reference									
No treatment to SABA	1.02(0.57)	0.07	0.86(0.57)	0.13	0.77(0.64)	0.22	1.24(0.71)	0.08	1.33(0.69)	0.06
No treatment, SABAtoICS	-0.06(0.19)	0.76	-0.03(0.19)	0.87	-0.19(0.21)	0.36	-0.08(0.24)	0.72	0.03(0.23)	0.088
Treated or not treated to OCS	-0.04(0.57)	0.94	-0.18(0.57)	0.74	-0.67(0.65)	0.29	0.11(0.72)	0.87	0.75(0.69)	0.28
Step down ICS/LTRA	0.02(0.24)	0.94	0.03(0.23)	0.91	0.04(0.26)	0.88	0.07(0.29)	0.79	-0.17(0.29)	0.54
Stop OCS	-0.65(0.25)	0.009	-0.56(0.25)	0.02	-1.02(0.28)	0.0002	-0.77(0.31)	0.01	-0.27(0.30)	0.37

S1.21 Effect of CRP (mg/L) on AQLQ and its dimensions

Parameters	Global AQLQ		AQLQ Symptom		AQLQ Activity		AQLQ Emotional		AQLQ Environmental	
	Estimate (std.error)	P-value	Estimate (std.error)	P-value	Estimate (std.error)	P-value	Estimate (std.error)	P-value	Estimate (std.error)	P-value
CRP (MG/L)	0.001(0.004)	0.77	-0.001(0.004)	0.95	-0003(0.005)	0.51	0.002(0.005)	0.64	0.006(0.005)	0.25
Time	0.57(0.07)	<0.0001	0.73(0.09)	<0.0001	0.56(0.09)	<0.0001	0.67(0.10)	<0.0001	0.19(0.09)	0.03
No change	Reference									
No treatment to SABA	1.04(0.58)	0.07	0.88(0.57)	0.12	0.76(0.65)	0.24	1.26(0.73)	0.08	1.33(0.7)	0.06
No treatment toICS	-0.06(0.19)	0.75	-0.03(0.19)	0.86	-0.22(0.22)	0.31	-0.11(0.25)	0.67	0.04(0.24)	0.86
Treated or not treated to OCS	-0.04(0.64)	0.94	-0.29(0.63)	0.64	-0.65(0.72)	0.37	0.02(0.81)	0.97	1.01(0.77)	0.19
Step down ICS/LTRA	-0.05(0.25)	0.84	-0.03(0.24)	0.89	-0.04(0.28)	0.89	0.02(0.31)	0.94	-0.27(0.29)	0.36
Stop OCS	-0.77(0.25)	0.002	-0.70(0.26)	0.006	-1.18(0.29)	<0.0001	-0.93(0.33)	0.005	-0.33(0.31)	0.28

Supplementary Table 2 (S2). Univariate binary logistic mixed model on binary AQLQ and its dimensions based on independent variables individually, with time and treatment changes group as fixed effect.

S2.1 Effect of ACT on binary AQLQ and its dimensions

Parameters	Global AQLQ		AQLQ Symptom		AQLQ Activity		AQLQ Emotional		AQLQ Environmental	
	Odds ratio	P-value	Odds ratio	P-value	Odds ratio	P-value	Odds ratio	P-value	Odds ratio	P-value
ACT	**1.789**	<0.0001	**1.618**	<0.0001	**1.499**	<0.0001	**1.489**	<0.0001	**1.266**	<0.0001
Time	1.115	0.756	1.845	0.064	0.683	0.179	0.876	0.647	0.598	0.040
No change	Reference									
No treatment to SABA	3.066	0.246	1.580	0.62	0.984	0.986	3.663	0.250	7.196	0.071
No treatment, SABAtoICS/LABA	0.581	0.186	0.920	0.829	1.374	0.361	0.634	0.281	1.187	0.635
Treated or not treated to OCS	1.548	0.752	0.369	0.487	1.402	0.788	2.351	0.550	3.667	0.250
Step down ICS/LTRA	0.493	0.164	0.638	0.355	1.352	0.483	1.054	0.918	0.718	0.472
Stop OCS	0.375	0.167	0.921	0.896	0.857	0.788	0.949	0.932	0.882	0.811

S2.2 Effect of age on binary AQLQ and its dimensions

Parameters	Global AQLQ		AQLQ Symptom		AQLQ Activity		AQLQ Emotional		AQLQ Environmental	
	Odds ratio	P-value	Odds ratio	P-value	Odds ratio	P-value	Odds ratio	P-value	Odds ratio	P-value
Age (Year)	1.019	0.525	1.015	0.164	0.996	0.704	1.006	0.567	**1.028**	0.013
Time	206.19	<0.0001	5.569	<0.0001	2.420	0.0004	2.816	<0.0001	1.281	0.280
No change	Reference									
No treatment to SABA	2.592	0.749	2.064	0.523	1.955	0.556	7.715	0.121	11.252	0.061
No treatment, SABAtoICS/LABA	0.690	0.722	0.901	0.799	1.232	0.596	0.667	0.370	1.214	0.641
Treated or not treated to OCS	1.316	0.928	0.383	.49	0.955	0.969	0.898	0.936	1.711	0.669
Step down ICS/LTRA	0.825	0.882	0.857	0.767	1.346	0.544	1.226	0.714	0.871	0.794
Stop OCS	0.229	0.393	0.323	0.065	0.301	0.043	0.267	0.035	0.367	0.085

S2.3 Effect of sex on binary AQLQ and its dimensions

Parameters	Global AQLQ		AQLQ Symptom		AQLQ Activity		AQLQ Emotional		AQLQ Environmental	
	Odds ratio	P-value	Odds ratio	P-value	Odds ratio	P-value	Odds ratio	P-value	Odds ratio	P-value
Sex	0.453	0.367	0.698	0.288	**0.326**	<0.0001	**0.418**	0.018	**0.380**	0.006
Time	205.16	<0.0001	5.653	<0.0001	2.431	<0.0001	2.841	<0.0001	1.326	0.219
No change	Reference									
No treatment to SABA	4.344	0.619	2.632	0.394	2.114	0.506	9.568	0.084	19.049	0.024
No treatment, SABAtoICS/LABA	0.651	0.682	0.878	0.751	1.167	0.688	0.639	0.317	1.144	0.745
Treated or not treated to OCS	1.288	0.934	0.412	0.524	0.856	0.894	0.882	0.925	1.879	0.612
Step down ICS/LTRA	0.915	0.946	0.843	0.742	1.531	0.376	1.299	0.634	0.882	0.812
Stop OCS	0.198	0.356	0.301	0.053	0.225	0.014	0.219	0.017	0.301	0.042

S2.4 Effect of BMI on binary AQLQ and its dimensions

Parameters	Global AQLQ		AQLQ Symptom		AQLQ Activity		AQLQ Emotional		AQLQ Environmental	
	**Odds ratio**	**P-value**	**Odds ratio**	**P-value**	**Odds ratio**	**P-value**	**Odds ratio**	**P-value**	**Odds ratio**	**P-value**
BMI (kg/m^2)	**0.879**	0.007	0.958	0.228	**0.889**	0.0007	0.968	0.399	0.949	0.136
Time	5.464	<0.0001	5.676	<0.0001	2.431	0.0004	2.851	<0.0001	1.326	0.219
No change	Reference									
No treatment to SABA	5.959	0.213	2.593	0.398	1.988	0.528	8.581	0.102	16.799	0.031
No treatment, SABAtoICS/LABA	0.668	0.417	0.918	0.843	1.356	0.429	0.683	0.399	1.241	0.606
Treated or not treated to OCS	1.394	0.816	0.443	0.558	1.081	0.946	0.979	0.988	2.103	0.550
Step down ICS/LTRA	0.673	0.522	0.792	0.654	1.288	0.594	1.187	0.757	0.767	0.618
Stop OCS	0.206	0.039	0.354	0.091	0.366	0.087	0.281	0.045	0.416	0.133

S2.5 Effect of smoking status on binary AQLQ and its dimensions

Parameters	Global AQLQ		AQLQ Symptom		AQLQ Activity		AQLQ Emotional		AQLQ Environmental	
	**Odds ratio**	**P-value**	**Odds ratio**	**P-value**	**Odds ratio**	**P-value**	**Odds ratio**	**P-value**	**Odds ratio**	**P-value**
Non-smokers	Reference									
Ex-Smokers	1.471	0.647	1.168	0.645	0.979	0.951	1.024	0.948	**2.233**	0.023
Current Smokers	0.145	0.373	0.348	0.063	0.871	0.761	0.666	0.433	**3.483**	0.009
Time	246.18	<0.001	5.548	<0.0001	2.387	0.0005	2.796	<0.001	1.365	0.178
No change	Reference									
No treatment to SABA	4.961	0.633	2.546	0.404	1.851	0.589	8.556	0.103	11.248	0.061
No treatment, SABAtoICS/LABA	0.702	0.742	0.926	0.851	1.236	0.591	0.667	0.371	1.107	0.805
Treated or not treated to OCS	1.625	0.882	0.439	0.554	0.924	0.947	0.935	0.961	1.677	0.671
Step down ICS/LTRA	0.783	0.855	0.804	0.669	1.337	0.553	1.161	0.787	0.828	0.717
Stop OCS	0.244	0.430	0.346	0.082	0.306	0.047	0.275	0.041	0.331	0.056

S2.6 Effect of atopy on binary AQLQ and its dimensions

Parameters	Global AQLQ		AQLQ Symptom		AQLQ Activity		AQLQ Emotional		AQLQ Environmental	
	Odds ratio	P-value	Odds ratio	P-value	Odds ratio	P-value	Odds ratio	P-value	Odds ratio	P-value
Atopy	2.341	0.372	2.039	0.482	**2.256**	0.046	1.168	0.681	0.567	0.121
Time	142.51	<0.0001	976.77	<0.0001	2.416	0.002	2.668	0.0005	1.232	0.406
No change	Reference									
No treatment to SABA	8.736	0.475	5.315	0.601	2.901	0.447	13.595	0.061	18.797	0.036
No treatment, SABAtoICS/LABA	1.151	0.905	1.615	0.715	1.446	0.462	1.004	0.994	1.574	0.337
Treated or not treated to OCS	2.319	0.788	8.146	0.960	0.955	0.974	1.331	0.838	2.636	0.467
Step down ICS/LTRA	1.231	0.887	1.555	0.784	1.074	0.909	1.310	0.662	0.788	0.695
Stop OCS	0.351	0.591	0.621	0.810	0.222	0.052	0.282	0.729	0.259	0.051

S2.7 Effect of onset of asthma on binary AQLQ and its dimensions

Parameters	Global AQLQ		AQLQ Symptom		AQLQ Activity		AQLQ Emotional		AQLQ Environmental	
	Odds ratio	P-value	Odds ratio	P-value	Odds ratio	P-value	Odds ratio	P-value	Odds ratio	P-value
Onset of asthma	1.013	0.677	0.976		1.002	0.909			**1.023**	0.004
Time	14.305	0.006	39.409		3.333	0.034			1.122	0.689
No change	Reference									
No treatment to SABA	16889.1	0.0001	333.3		1368.38	0.566			15.138	0.068
No treatment, SABAtoICS/LABA	0.651	0.783	2.009		0.737	0.765			0.959	0.918
Treated or not treated to OCS	0.00001	0.999	0.00003		0.0001	0.989			0.701	0.805
Step down ICS/LTRA	0.921	0.964	0.883		0.949	0.971			0.946	0.912
Stop OCS	0.214	0.675	0.000001		0.256	0.523			0.389	0.150

S2.8 Effect of FeNO on binary AQLQ and its dimensions

Parameters	Global AQLQ		AQLQ Symptom		AQLQ Activity		AQLQ Emotional		AQLQ Environmental	
	**Odds ratio**	**P-value**	**Odds ratio**	**P-value**	**Odds ratio**	**P-value**	**Odds ratio**	**P-value**	**Odds ratio**	**P-value**
FENO (ppb)	0.991	0.362	1.001	0.765	**1.007**	0.031	1.001	0.823	1.002	0.558
Time	349.57	<0.0001	5.752	<0.0001	2.794	<0.0001	2.931	<0.0001	1.404	0.147
No change	Reference									
No treatment to SABA	2.702	0.737	2.449	0.419	1.952	0.548	7.741	0.107	15.684	0.033
No treatment, SABAtoICS/LABA	0.741	0.786	0.876	0.745	1.151	0.721	0.662	0.348	1.161	0.721
Treated or not treated to OCS	1.179	0.958	0.406	0.514	0.935	0.954	0.911	0.943	1.924	0.598
Step down ICS/LTRA	0.748	0.836	0.783	0.634	1.192	0.717	1.104	0.8552	0.731	0.551
Stop OCS	0.254	0.452	0.313	0.057	0.259	0.025	0.264	0.030	0.362	0.081

S2.9 Effect of sputum neutrophils on binary AQLQ and its dimensions

Parameters	Global AQLQ		AQLQ Symptom		AQLQ Activity		AQLQ Emotional		AQLQ Environmental	
	**Odds ratio**	**P-value**	**Odds ratio**	**P-value**	**Odds ratio**	**P-value**	**Odds ratio**	**P-value**	**Odds ratio**	**P-value**
Sputum neutrophils (10^3/g)	1.034	0.056	1.001	0.969	0.994	0.342	**1.013**	0.042	0.999	0.934
Time	218.95	<0.0001	5.835	<0.0001	2.787	0.0004	2.638	0.0008	1.425	0.191
No change	Reference									
No treatment to SABA	167.56	0.363	5.717	0.209	3.377	0.343	16.009	0.068	19.775	0.063
No treatment ICS/LABA	0.476	0.578	0.747	0.552	0.917	0.842	0.793	0.607	1.032	0.945
Treated or not treated to OCS	0.846	0.961	0.342	0.475	0.773	0.833	0.898	0.932	2.038	0.586
Step down ICS/LTRA	0.485	0.662	0.458	0.231	1.087	0.745	1.242	0.691	0.661	0.473
Stop OCS	0.136	0.401	0.310	0.119	0.198	0.022	0.342	0.096	0.299	0.073

S2.10 Effect of sputum neutrophils on binary AQLQ and its dimensions

Parameters	Global AQLQ		AQLQ Symptom		AQLQ Activity		AQLQ Emotional		AQLQ Environmental	
	**Odds ratio**	**P-value**	**Odds ratio**	**P-value**	**Odds ratio**	**P-value**	**Odds ratio**	**P-value**	**Odds ratio**	**P-value**
Sputum eosinophils (10^3/g)	**0.926**	0.026	**0.905**	0.008	0.987	0.136	**0.971**	0.003	0.985	0.082
Time	77.57	<0.0001	242.18	<0.001	2.502	0.002	2.392	0.002	1.292	0.345
No change	Reference									
No treatment to SABA	128.49	0.379	11.351	0.747	2.827	0.425	13.863	0.078	17.225	0.075
No treatment, SABA to ICS/LABA	0.599	0.690	0.876	0.921	1.009	0.982	0.826	0.669	1.105	0.831
Treated or not treated to OCS	1.448	0.921	0.391	0.834	0.764	0.829	1.041	0.974	2.121	0.571
Step down ICS/LTRA	0.463	0.624	0.361	0.561	1.067	0.904	1.135	0.814	0.628	0.426
Stop OCS	0.196	0.461	0.421	0.667	0.210	0.024	0.364	0.114	0.308	0.081

S2.11 Effect of blood neutrophils on binary AQLQ and its dimensions

Parameters	Global AQLQ		AQLQ Symptom		AQLQ Activity		AQLQ Emotional		AQLQ Environmental	
	**Odds ratio**	**P-value**	**Odds ratio**	**P-value**	**Odds ratio**	**P-value**	**Odds ratio**	**P-value**	**Odds ratio**	**P-value**
Blood neutrophils (μL)	0.992	0.823	1.011	0.555	0.978	0.144	1.001	0.936	1.027	0.068
Time	174.59	<0.001	6.383	<0.0001	2.622	0.0002	2.895	<0.0001	1.325	0.228
No change	Reference									
No treatment to SABA	3.271	0.688	2.565	0.439	1.831	0.601	8.716	0.112	16.186	0.034
No treatment to ICS/LABA	0.687	0.717	0.851	0.715	1.151	0.728	0.623	0.317	1.197	0.667
Treated or not treated to OCS	1.399	0.912	0.337	0.466	1.128	0.921	0.889	0.934	1.502	0.745
Step down ICS/LTRA	0.765	0.839	0.731	0.578	1.371	0.531	1.126	0.838	0.713	0.525
Stop OCS	0.237	0.399	0.306	0.070	0.288	0.041	0.262	0.04	0.366	0.085

S2.12 Effect of blood eosinophils on binary AQLQ and its dimensions

Parameters	Global AQLQ		AQLQ Symptom		AQLQ Activity		AQLQ Emotional		AQLQ Environmental	
	Odds ratio	P-value	Odds ratio	P-value	Odds ratio	P-value	Odds ratio	P-value	Odds ratio	P-value
Blood eosinophils (µL)	**0.717**	0.007	**0.776**	0.009	0.923	0.075	**0.840**	0.003	0.971	0.433
Time	80.725	<0.0001	6.755	0.0002	2.441	0.0006	2.723	<0.001	1.321	0.238
No change	Reference									
No treatment to SABA	3.214	0.716	2.635	0.496	1.756	0.626	9.077	0.133	16.364	0.035
No treatment to ICS/LABA	0.692	0.735	0.941	0.904	1.253	0.588	0.667	0.423	1.174	0.709
Treated or not treated to OCS	0.696	0.905	0.255	0.417	0.861	0.905	0.802	0.883	1.945	0.603
Step down ICS/LTRA	0.501	0.611	0.631	0.486	1.311	0.601	1.037	0.951	0.725	0.555
Stop OCS	0.211	0.349	0.281	0.101	0.294	0.048	0.251	0.046	0.375	0.098

S2.13 Effect of total IgE on binary AQLQ and its dimensions

Parameters	Global AQLQ		AQLQ Symptom		AQLQ Activity		AQLQ Emotional		AQLQ Environmental	
	Odds ratio	P-value	Odds ratio	P-value	Odds ratio	P-value	Odds ratio	P-value	Odds ratio	P-value
Total IgE (KU/L)	1.001	0.396	1.001	0.344	1.001	0.375	1.001	0.529	0.999	0.729
Time	38.483	<0.001	6.426	<0.0001	2.445	0.001	2.551	0.0007	1.363	0.238
No change	Reference									
No treatment to SABA	4.986	0.605	3.401	0.327	2.042	0.551	8.596	0.097	22.021	0.41
No treatment to ICS	0.811	0.846	1.071	0.888	1.342	0.511	0.724	0.493	1.194	0.723
Treated to OCS	1.502	0.886	0.498	0.640	0.996	0.998	1.016	0.991	2.122	0.598
Step down ICS/LTRA	0.703	0.802	0.709	0.594	1.151	0.802	1.044	0.91	0.875	0.831
Stop OCS	0.346	0.521	0.426	0.234	0.375	0.135	0.342	0.101	0.274	0.067

S2.14 Effect of fibrinogen on binary AQLQ and its dimensions

Parameters	Global AQLQ		AQLQ Symptom		AQLQ Activity		AQLQ Emotional		AQLQ Environmental	
	Odds ratio	P-value	Odds ratio	P-value	Odds ratio	P-value	Odds ratio	P-value	Odds ratio	P-value
Fibrinogen (g/l)	0.491	0.158	0.756	0.201	0.668	0.051	1.019	0.926	1.217	0.276
Time	136.56	<0.0001	6.508	<0.0001	2.876	0.0001	2.991	0.0001	1.571	0.066
No change	Reference									
No treatment to SABA	2.720	0.746	2.894	0.364	1.505	0.734	9.057	0.105	13.093	0.041
No treatment, SABA to ICS/LABA	73.05	0.793	1.207	0.683	1.211	0.664	0.788	0.626	1.042	0.922
Treated or not treated to OCS	1.547	0.901	0.654	0.771	1.072	0.956	1.229	0.883	2.056	0.559
Step down ICS/LTRA	0.767	0.856	1.018	0.975	1.174	0.771	1.466	0.527	0.762	0.612
Stop OCS	0.214	0.447	0.327	0.111	0.228	0.030	0.251	0.043	0.215	0.015

S2.15 Effect of FEV1 pre (%) on binary AQLQ and its dimensions

Parameters	Global AQLQ		AQLQ Symptom		AQLQ Activity		AQLQ Emotional		AQLQ Environmental	
	**Odds ratio**	**P-value**	**Odds ratio**	**P-value**	**Odds ratio**	**P-value**	**Odds ratio**	**P-value**	**Odds ratio**	**P-value**
FEV1 pre (%)	**1.081**	0.003	**1.027**	0.003	**1.046**	<0.0001	**1.035**	<0.0001	**1.031**	0.0001
Time	109.58	<0.0001	5.802	<0.001	2.288	0.002	2.681	0.0001	1.207	0.417
No change	Reference									
No treatment to SABA	2.813	0.736	1.913	0.588	1.108	0.928	5.448	0.194	10.986	0.061
No treatment to ICS/LABA	0.681	0.745	0.933	0.874	1.357	0.451	0.703	0.435	1.306	0.522
Treated or not treated to OCS	7.235	0.566	0.464	0.614	1.284	0.844	1.246	0.875	2.878	0.411
Step down ICS/LTRA	0.806	0.884	0.779	0.652	1.271	0.633	1.132	0.824	0.772	0.624
Stop OCS	0.369	0.594	0.405	0.162	0.396	0.135	0.346	0.092	0.504	0.237

S2.16 Effect of FEV1 post (%) on binary AQLQ and its dimensions

Parameters	Global AQLQ		AQLQ Symptom		AQLQ Activity		AQLQ Emotional		AQLQ Environmental	
	**Odds ratio**	**P-value**	**Odds ratio**	**P-value**	**Odds ratio**	**P-value**	**Odds ratio**	**P-value**	**Odds ratio**	**P-value**
FEV1 post (%)	**1.051**	0.023	**1.023**	0.009	**1.042**	<0.0001	**1.029**	0.0006	**1.028**	0.0004
Time	128.81	<0.0001	5.983	<0.0001	2.463	<0.0001	2.858	<0.0001	1.302	0.256
No change	Reference									
No treatment to SABA	3.393	0.678	1.973	0.568	1.113	0.926	5.902	0.177	11.618	0.058
No treatment to ICS/LABA	0.793	0.832	0.942	0.890	1.367	0.439	0.713	0.455	1.348	0.4799
Treated or not treated to OCS	2.735	0.753	0.432	0.574	1.112	0.932	1.083	0.954	2.548	0.468
Step down ICS/LTRA	0.874	0.921	0.796	0.677	1.314	0.586	1.176	0.771	0.794	0.665
Stop OCS	0.383	0.585	0.401	0.155	0.405	0.143	0.342	0.091	0.511	0.251

S2.17 Effect of FVC pre (%) on binary AQLQ and its dimensions

Parameters	Global AQLQ		AQLQ Symptom		AQLQ Activity		AQLQ Emotional		AQLQ Environmental	
	Odds ratio	P-value	Odds ratio	P-value	Odds ratio	P-value	Odds ratio	P-value	Odds ratio	P-value
FVC pre (%)	**1.066**	0.009	**1.025**	0.013	**1.048**	<0.0001	**1.034**	0.0005	**1.029**	0.0006
Time	165.95	<0.0001	6.294	<.0001	2.582	<0.0001	2.972	<0.0001	1.321	0.229
No change	Reference									
No treatment to SABA	5.653	0.561	2.115	0.538	1.243	0.849	6.794	0.151	13.344	0.046
No treatment to ICS/LABA	0.848	0.883	0.953	0.912	1.458	0.344	0.727	0.484	1.349	0.473
Treated or not treated to OCS	3.853	0.674	0.416	0.559	1.074	0.953	1.038	0.978	2.361	0.497
Step down ICS/LTRA	0.811	0.881	0.762	0.623	1.219	0.686	1.104	0.861	0.754	0.592
Stop OCS	0.428	0.638	0.386	0.143	0.431	0.164	0.338	0.088	0.501	0.232

S2.18 Effect of FVC post (%) on binary AQLQ and its dimensions

Parameters	Global AQLQ		AQLQ Symptom		AQLQ Activity		AQLQ Emotional		AQLQ Environmental	
	**Odds ratio**	**P-value**	**Odds ratio**	**P-value**	**Odds ratio**	**P-value**	**Odds ratio**	**P-value**	**Odds ratio**	**P-value**
FVC post (%)	1.043	0.059	**1.022**	0.028	**1.038**	<0.0001	**1.027**	0.005	**1.028**	0.002
Time	1.794	<0.0001	6.309	<0.0001	2.574	<0.001	3.036	<0.0001	1.377	0.168
No change	Reference									
No treatment to SABA	4.606	0.604	2.0001	0.564	1.166	0.891	6.513	0.158	12.44	0.052
No treatment to ICS/LABA	7.757	0.812	0.915	0.838	1.332	0.461	0.694	0.421	1.311	0.516
Treated or not treated to OCS	1.732	0.858	0.371	0.501	0.861	0.898	0.869	0.918	1.972	0.588
Step down ICS/LTRA	7.743	0.848	0.753	0.605	1.232	0.663	1.115	0.845	0.754	0.591
Stop OCS	0.328	0.524	0.365	0.118	0.393	0.115	0.316	0.069	0.482	0.206

S2.19 Effect of FEV1/FVC pre on binary AQLQ and its dimensions

Parameters	Global AQLQ		AQLQ Symptom		AQLQ Activity		AQLQ Emotional		AQLQ Environmental	
	**Odds ratio**	**P-value**	**Odds ratio**	**P-value**	**Odds ratio**	**P-value**	**Odds ratio**	**P-value**	**Odds ratio**	**P-value**
FEV1/FVC pre	1.081	0.059	1.021	0.165	**1.035**	0.018	**1.035**	0.031	1.021	0.142
Time	146.84	<0.0001	5.511	<0.0001	2.293	0.001	2.683	0.0001	1.267	0.305
No change	Reference									
No treatment to SABA	2.401	0.775	2.481	0.428	1.758	0.624	7.731	0.118	15.253	0.036
No treatment, SABA to ICS	0.698	0.741	0.889	0.778	1.231	0.606	0.661	0.365	1.201	0.663
Treated or not treated to OCS	2.494	0.779	0.464	0.593	1.178	0.894	1.189	0.899	2.394	0.494
Step down ICS/LTRA	0.981	0.989	0.829	0.723	1.424	0.481	1.255	0.686	0.818	0.706
Stop OCS	0.265	0.459	0.349	0.091	0.317	0.059	0.291	0.052	0.405	0.123

S2.20 Effect of FEV1/FVC post on binary AQLQ and its dimensions

Parameters	Global AQLQ		AQLQ Symptom		AQLQ Activity		AQLQ Emotional		AQLQ Environmental	
	**Odds ratio**	**P-value**	**Odds ratio**	**P-value**	**Odds ratio**	**P-value**	**Odds ratio**	**P-value**	**Odds ratio**	**P-value**
FEV1/FVC post	1.048	0.194	1.016	0.281	**1.042**	0.006	**1.034**	0.031	1.015	0.282
Time	1.508	<0.0001	5.567	<0.0001	2.289	0.001	2.683	0.0001	1.288	0.269
No change	Reference									
No treatment to SABA	2.865	0.725	2.563	0.413	1.863	0.593	7.731	0.118	15.324	0.033
No treatment, SABA to ICS	0.719	0.753	0.883	0.766	1.221	0.625	0.661	0.365	1.206	0.651
Treated or not treated to OCS	2.113	0.812	0.456	0.583	1.249	0.858	1.189	0.899	2.258	0.515
Step down ICS/LTRA	0.936	0.960	0.824	0.715	1.426	0.484	1.256	0.686	0.812	0.693
Stop OCS	0.286	0.469	0.348	0.091	0.326	0.069	0.291	0.052	0.411	0.125

S2.21 Effect of CRP (mg/L) on binary AQLQ and its dimensions

Parameters	Global AQLQ		AQLQ Symptom		AQLQ Activity		AQLQ Emotional		AQLQ Environmental	
	Odds ratio	P-value	Odds ratio	P-value	Odds ratio	P-value	Odds ratio	P-value	Odds ratio	P-value
CRP (MG/L)	1.006	0.645	1.006	0.631	0.996	0.768	1.004	0.767	1.021	0.079
Time	5.163	0.0001	5.868	<0.0001	2.938	<0.001	3.057	<0.0001	1.688	0.035
No change	Reference									
No treatment to SABA	5.019	0.254	2.563	0.395	1.587	0.685	8.591	0.109	15.246	0.037
No treatment to ICS	0.656	0.408	0.918	0.842	1.139	0.753	0.728	0.504	1.152	0.744
Treated or not treated to OCS	1.704	0724	0.574	0.701	1.208	0.881	1.671	0.728	3.420	0.371
Step down ICS/LTRA	0.660	0.510	0.825	0.718	1.147	0.756	1.259	0.692	0.596	0.346
Stop OCS	0.144	0.017	0.269	0.049	0.219	0.018	0.231	0.029	0.288	0.043
